# Cardiac and Obstetric Complications of Pregnant Women with Rheumatic Heart Disease in Sub-Saharan Africa: A Systematic Review

**DOI:** 10.5334/gh.1522

**Published:** 2026-01-30

**Authors:** Evangelia Alexopoulos, Doreen Nakagaayi, Elizabeth R. Blackwood, Felix Barasa, Joan Kiyeng, Wycliffe Kosgei, G. Titus Ng’eno, Shanti Nulu, Rebecca Lumsden, Andrea Beaton, Gerald S. Bloomfield

**Affiliations:** 1 Duke University School of Medicine, Durham, NC, US; 2 Department of Adult Cardiology, Uganda Heart Institute, Kampala, UG; 3 Department of Internal Medicine, Makerere University College of Health Sciences, Kampala, UG; 4 Duke University Medical Center Library & Archives, Durham, NC, US; 5 Department of Cardiology, Moi Teaching and Referral Hospital, Eldoret, KE; 6 Department of Obstetrics and Gynecology, Moi Teaching and Referral Hospital, Eldoret, KE; 7 Duke Global Health Institute, Duke University, Durham, NC, US; 8 Division of Cardiology, Department of Medicine, Duke University School of Medicine, Durham, NC, US; 9 Division of Cardiology, Department of Internal Medicine, The University of Texas at Austin Dell Medical School, Austin, TX, US; 10 Department of Medicine, Duke University School of Medicine, Durham, NC, US; 11 Cincinnati Children’s Hospital Medical Center, Cincinnati, OH, US; 12 University of Cincinnati School of Medicine, Cincinnati, OH, US; 13 Duke Clinical Research Institute, Duke University, Durham, NC, US

**Keywords:** rheumatic heart disease, pregnancy, SSA, cardiac outcomes, heart failure, obstetric outcomes

## Abstract

**Background:**

Rheumatic heart disease (RHD) is a key contributor to maternal cardiovascular morbidity and mortality in sub-Saharan Africa (SSA). Though low- and middle-income countries (LMICs), particularly those in SSA, face a greater burden of RHD, existing systematic reviews have not specifically focused on cardiac and obstetric complications among affected women. We aimed to study cardiac and obstetric complications in pregnant and postpartum women with RHD in SSA and to evaluate the rate of valvular interventions in pregnant or postpartum women with severe disease.

**Methods:**

We performed a systematic search in MEDLINE and online sources for studies of women of childbearing age (15–49 years) with RHD published after 2000 in SSA. Included study types were randomized controlled trials, retrospective and prospective cohort studies, case-control studies, case reports, and case series. Two authors independently extracted data and critically appraised articles. PROSPERO registration number: CRD42024628121.

**Results:**

We identified 1,478 unique citations, and nine full-text studies met inclusion criteria. Included studies were case series (7), one cohort study, and one case-control study, including a total of 787 pregnant women with cardiac disease, of whom the majority had RHD. Mitral stenosis and regurgitation were the most common valve lesions. Heart failure and arrhythmia occurred in at least 12.9% and up to 36% of study participants, respectively. Eight studies reported deaths due to cardiac causes (median: six deaths due to cardiac disease; total number of deaths: 56). Preterm labor/delivery was the most reported obstetric event, with incidence ranging from 5.2–35.2%. Few pregnant patients received any valve intervention.

**Conclusions:**

Pregnant women with RHD in SSA are at risk for both adverse cardiac and obstetric outcomes in pregnancy, particularly heart failure and preterm labor. Future efforts may include registries focused on pregnant women with RHD and scaling cardiac interventional capacity to benefit pregnant women with RHD in SSA.

**Unstructured Abstract:**

We performed a systematic search in MEDLINE and online sources to study cardiac and obstetric complications and rates of valvular interventions in pregnant and postpartum women with rheumatic heart disease (RHD) in sub-Saharan Africa (SSA). Two authors independently extracted data and critically appraised articles. Nine full-text studies met inclusion criteria, capturing 787 pregnant women with cardiac disease, mostly RHD. Heart failure and arrhythmia occurred in at least 12.9% and up to 36% of study participants, respectively. Fifty-six deaths were reported from cardiac causes. Preterm labor/delivery was the most reported obstetric event, and few pregnant patients received any valve intervention. We found that women with RHD in SSA are at risk for adverse cardiac and obstetric outcomes in pregnancy, particularly heart failure and preterm labor. Future efforts may include registries focused on pregnant women with RHD and scaling cardiac interventional capacity to benefit pregnant women with RHD in SSA.

## Introduction

Rheumatic heart disease (RHD) affects nearly 55 million people worldwide, leading to at least 360,000 mortalities annually (1,2). RHD is a leading cause of cardiac morbidity in sub-Saharan Africa (SSA), with this region carrying 23% of the world’s prevalent RHD cases (3). Significant global disparities exist in the management of advanced RHD, as high-income countries (HICs) have greater primary prevention strategies for identification and treatment of childhood streptococcal infections and higher rates of surgical correction of valvular lesions (3,4,5,6,7). With limited rollout of these strategies, low- and middle-income countries (LMICs) bear higher rates of mortality and complications, such as heart failure, arrhythmia, thromboembolism, and infective endocarditis, due to RHD (8). While RHD has largely been eliminated in HICs due to socioeconomic advantages and early access to diagnosis and treatment, RHD extensively impacts many LMICs in sub-Saharan Africa (SSA) (9,10).

The majority of patients with RHD are women of reproductive age (9,11). Pregnancy with RHD, especially advanced RHD, has potentially severe consequences related to both maternal and fetal well-being (12). RHD severity is defined by several factors, utilizing echocardiographic-based parameters (such as ejection fraction, valve area, or gradient) and hemodynamic factors, including the presence of pulmonary hypertension and functional assessment with the New York Heart Association (NYHA) class (13,14). Normal physiologic changes in pregnancy can unmask previously undiagnosed valvular disease, especially mitral stenosis (MS), as significant changes in cardiac output, systemic vascular resistance, and procoagulant factors occur throughout pregnancy and delivery (15). Heart failure is a commonly reported cardiac complication (16). Right-sided heart failure can occur with severe MS and pulmonary hypertension, and left-sided heart failure can occur with severe mitral or aortic regurgitation (AR). Other common complications of RHD include atrial fibrillation and stroke. Valve regurgitation may predispose patients to volume overload and atrial arrhythmias during pregnancy (13). Obstetric outcomes such as preterm delivery and fetal outcomes such as low birth weight have been observed in individuals with RHD (13,17,18).

Valvular interventions such as percutaneous balloon mitral valvuloplasty for MS have been established to be safe during pregnancy and to reduce symptoms and fetal complications (19). Surgery is indicated for severe, symptomatic valvulopathy in RHD, and percutaneous balloon mitral valvuloplasty is indicated for severe isolated MS (20,21). However, progressive valve worsening, especially in younger patients, hospital capacity, and surgeon skill set and confidence pose persistent challenges in resource-limited settings (22). Open heart surgeries are performed at a rate of two per million in SSA, and it is unclear how many women receive operations for RHD during pregnancy in this region. Outside of South Africa, there are only 22 cardiac surgical centers in SSA, one per 33 million people (23). In comparison, it is estimated that there is one center per 120,000 people in the United States (24).

RHD disproportionately affects LMICs and significantly impacts maternal outcomes and both direct and indirect mortality in SSA (25,26,27). Despite this known burden, existing systematic reviews have not focused on cardiac and obstetric complications in pregnant women with RHD in SSA. To address this shortcoming in the literature, we aimed to explore pregnancy as an exposure of interest for cardiac and obstetric complications in pregnant or postpartum women with RHD in SSA and to evaluate the number of women with severe disease who receive cardiac intervention during pregnancy or in the postpartum period.

## Methods

### Study objectives

Our study aimed to address several questions about the cardiovascular and obstetric risks surrounding pregnancy in women with RHD in SSA. We studied the rates of cardiac and obstetric complications in pregnant or postpartum women with RHD. We also investigated how many pregnant women with severe RHD received cardiac intervention during pregnancy or within 12 months of delivery.

### Study protocol and registration

This review followed the PRISMA guideline for systematic reviews (28) and was registered in the International Prospective Register of Systematic Reviews (PROSPERO) database under the registration number CRD42024628121.

### Search strategy and information sources

The search was developed and conducted by a professional medical librarian in consultation with the author team and included a mix of keywords and subject headings representing pregnancy, RHD, and LMICs. Search hedges or database filters were used to remove publication types such as editorials, letters, notes, and conference abstracts as was appropriate for each database. Searches were conducted in MEDLINE via PubMed, Embase via Elsevier, Web of Science via Clarivate, Global Health via EBSCOhost, and Global Index Medicus via the World Health Organization (WHO). The searches were executed on December 16, 2024, and found 1,478 unique citations. Complete reproducible search strategies, including search filters, for all databases are detailed in the Supplemental Materials (Supplemental Table 1). All citations were imported into Covidence, a systematic review screening software, which also deduplicates the results.

### Inclusion/exclusion criteria

Inclusion criteria included studies of women of child-bearing age (15–49) with a diagnosis of RHD living in SSA; study types included were randomized controlled trials (RCTs), non-randomized observational studies including retrospective and prospective cohort studies, case-control studies, case reports, and case series. English-language studies in the year 2000 or later were included. Case reports were included in the initial search to obtain useful references; however, data was not extracted from case reports, and these studies were ultimately excluded. We included studies that reported combined outcomes for pregnant women with cardiac disease if at least 60% of the study population consisted of participants with RHD, and we excluded studies in which less than 60% of participants had RHD. We excluded studies focused solely on congenital or degenerative heart disease.

### Study selection

Abstract review of records was performed by two investigators (EA, GSB). Each author reviewed the titles and abstracts independently to determine if they should be included. In cases of disagreement, the record was revisited to reach a consensus.

### Data extraction

A Google Form was developed for authors to use as a tool for data extraction. Data collection was performed by two authors (EA, DN). The key data were predetermined and included title, first author’s name, year of publication, study design, country of origin, study setting (hospital-based, community-based, or combined), total sample size, number of women of reproductive age (15–49) with RHD, type of valvular lesion and if severe disease is present, and number of women with RHD currently pregnant or who delivered within the last year. Of this sample of pregnant or recently pregnant women with RHD, we collected: total number of any cardiac complication as determined by the individual study, hospitalization for heart failure, arrhythmia (atrial or ventricular), infective endocarditis, stroke, venous or arterial thromboembolism, valvular complication (complication following valvular intervention), acute coronary syndrome or death due to cardiovascular disease, total number of any obstetric complication as determined by the individual study, spontaneous pregnancy loss (including miscarriage, first and second trimester losses, and intra-uterine fetal demise and excluding terminations), newborn/neonatal death, maternal death, preterm delivery, preeclampsia, intrauterine growth restriction (IUGR) or low birth weight, postpartum hemorrhage, rates of cardiac intervention including valve replacement, valve repair/valvuloplasty/commissurotomy, valve myotomy, left atria reduction, left atrial appendage exclusion (clip/stich), and Maze procedure. Definitions for early pregnancy loss came from the American College of Obstetricians and Gynecologists. Definitions for newborn/neonatal death, maternal death, preterm delivery, and intrauterine growth restriction were obtained from the WHO. Maternal death was defined by the WHO as the death of a woman while pregnant or within 42 days of termination of pregnancy, irrespective of the duration and site of the pregnancy, from any cause related to or aggravated by the pregnancy or its management but not from accidental or incidental causes (29). This is an aggregate number that included death due to maternal cardiac causes.

### Quality assessment

Two authors (EA, DN) independently assessed the quality of the studies using a Joanna Briggs Institute (JBI) critical appraisal tool (30,31). Consensus was reached for each checklist item. The appraisal tools were specific to the study type (i.e., case series, cohort study, and case-control study).

### Data synthesis

Data was combined in a narrative format due to a low volume of studies of varying quality and to mitigate potential issues of data completeness. There was a large amount of variability between the specific data points collected by each study.

## Results

**Figure 1:**
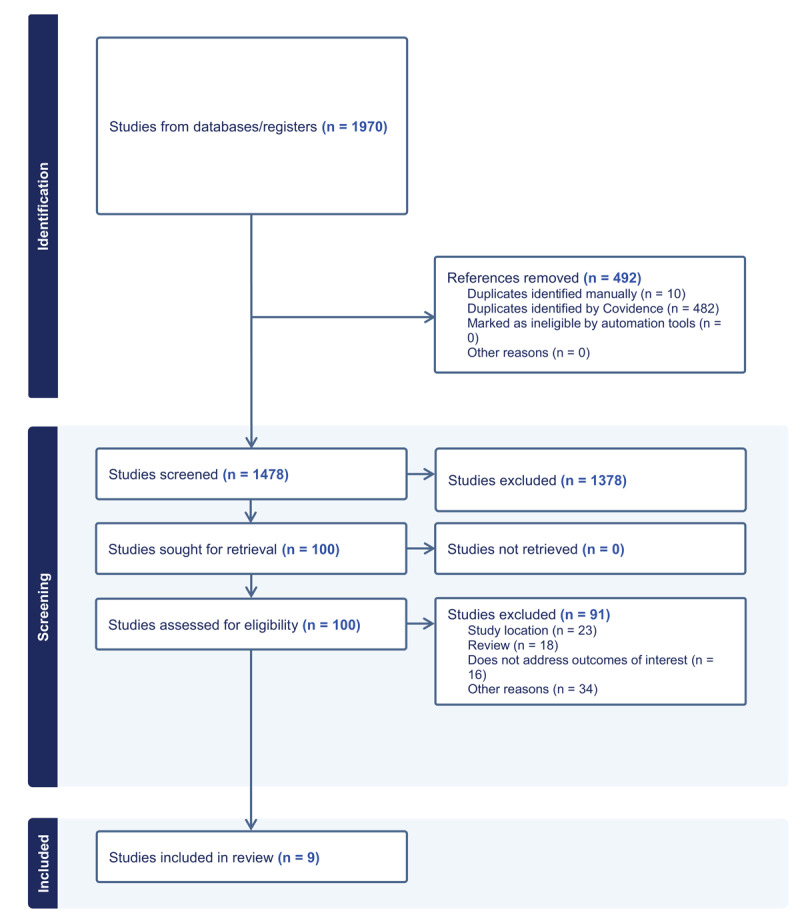
Study flow diagram.

Our search resulted in 1,478 unique citations, of which 1,378 abstracts were excluded (Figure 1). One hundred full-text studies were reviewed, and nine were selected for inclusion. We extracted our outcomes of interest for each study.

### Study characteristics

Study characteristics are summarized in Table 1. These studies were published between 2000 and 2022 and represented 4,477 individuals. Seven case series, one cohort study, and one case-control study were included. One of the initial objectives of this systematic review was to compare pregnant and non-pregnant age-matched women with RHD; however, we did not find any studies that compared these two groups. All the studies were of pregnant women with cardiac disease, mostly RHD. We included two studies that reported on procedural interventions for pregnant women with RHD (32,33).

**Table 1:** Study characteristics.


AUTHOR (YEAR)	COUNTRY	STUDY DESIGN	SAMPLE SIZE	SAMPLE WITH RHD, n (%)	AGE DIST. (YEARS)	FINDINGS/COMMENTS

*Beaton et al. (2019) (34)	Uganda	Prospective cohort	3506 (58 with cardiac disease)	51 (87.9%)	*Participants with cardiac disease*:Median 29.5 (IQR: 24, 35)	Two health centers and one referral hospital Echo was used to classify patients with heart disease (N = 58) and without heart disease (N = 3,448) Cardiovascular complications occurred in over half of women with heart disease, most commonly heart failure

**Desai et al. (2000)** (33)	South Africa	Case series	128	128 (100%)	Mean: 27 (Range: 16–44)	Mitral valve stenosis only Doppler-echo used for measurement of MVA Half (49%) had at least moderately severe disease Balloon valvulotomy performed in 20 individuals

*Diao et al. (2011) (38)	Senegal	Case series	50	46 (92%)	Mean: 28.4 (Range: 18–43)	32 participants with MS 36 with pulmonary edema at admission All participants required cardiovascular medication and three required cardioversions High mortality rate (17 deaths), 11 postpartum

**Gebremedhin et al. (2022)** (47)	Ethiopia	Case series	58	58 (100%)	Mean: 27 (SD: 4.5)	MS only Echo used for measurement of mitral valve area 67% of participants had severe pulmonary hypertension Most participants (69%) had heart failure and pulmonary edema

*Hailu et al. (2019) (39)	Ethiopia	Case series	21	20 (95.2%)	Mean: 26.7 (SD: 5.7)	Study of pregnant patients admitted with pulmonary hypertension (measured by echo); mean pulmonary arterial pressure was 102.9 (SD 16.9) Heart failure occurred in all patients Preeclampsia occurred in five patients

*Lumsden et al. (2020) (37)	Kenya	Case-control study	339 (97 cardiac cases and 242 controls)	73 (75.3%)	*Participants with cardiac disease*: Median: 26 (Range: 16–50)	Mitral regurgitation present in 45 patients; MS present in 40 participants (over half of those were severe) 61.4% NYHA Class III or IV on admission Higher rates of adverse events occurred than were predicted by risk scores Mortality in pregnant women with cardiac conditions was nearly 10 times higher than all-cause mortality among pregnant women

*Nqayana et al. (2008) (32)	South Africa	Case series	95	77 (81.1%)	72 (75.8%) < 30 years 54% ≤25 years	20 individuals had valve replacement prior to pregnancy Eight required balloon valvuloplasty during pregnancy No maternal deaths in study period Heart failure was most common cardiac complication, occurring in 13 individuals

*Poli et al. (2020) (40)	Kenya	Case series	91	80 (87.9%)	Median: 27 (IQR: 23, 33)	Heart failure, pulmonary edema, and arrhythmias were the most common cardiac adverse outcomes Investigated sociodemographic factors and risk of adverse outcomes After adjustment, maternal age, marital status, occupation, and residence were not associated with adverse outcomes; low education level increased odds of adverse outcomes 3.3-fold (p = 0.049)

*Soma-Pillay et al. (2008) (41)	South Africa	Case series	189	120 (63.5%)	Mean: 27.34 (SD: 6.7)	Investigated ‘near miss’ data, acute organ system dysfunction that could result in death Found 25 near misses Pulmonary edema was the cause of four maternal deaths


*Study consisted of participants with RHD and other cardiac disease, including congenital disease and peripartum cardiomyopathy. MS: mitral stenosis; MVA: mitral valve area; NYHA: New York Heart Association.

Study sample sizes ranged from 21 to over 3,000 participants with a median of 95 study participants [Q1, Q3: 54, 264] (Table 1). Our nine studies included a total of 787 pregnant women with cardiac disease, of whom the majority or all (63.5% to 100%) study participants were pregnant women with RHD. All studies reported a mean or median age of study participants between 26 and 30 years old. Most (N = 8) studies were hospital-based; one was a compiled study of regional health centers and one hospital (34).

Detailed reasons for exclusion can be found in Supplemental Table 2. The most common reasons for exclusion were study location (many were outside of the study region), study type (review or case report), and reported outcomes that were not included in our review protocol. Many excluded studies were conducted in India, Australia, and Southeast Asia or did not include details for participants specifically from SSA in the case of international cohort studies (16,35). We excluded one study that treated patients from Portuguese-speaking African nations, as these patients decided to permanently relocate and receive their care in Portugal (36). We included seven studies in SSA that presented combined outcomes for patients with cardiac disease with RHD present in ≥60% of the study population; other cardiac diseases included congenital disease and peripartum cardiomyopathy (32,34,37,38,39,40,41). Of the seven studies with combined outcomes, five studies reported RHD in >80% of study participants (32,34,38,39,40). We excluded six studies with outcomes of interest in which pregnant women with RHD were <60% of the total study population (27,42,43,44,45,46).

**Figure 2:**
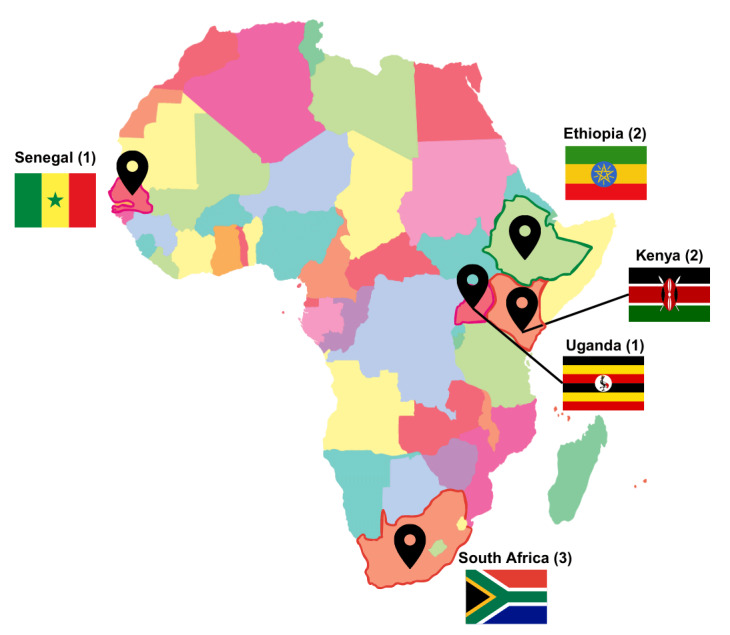
Geographic distribution of studies.

Included studies were conducted in South Africa (3), Kenya (2), Ethiopia (2), Senegal (1), and Uganda (1). Geographic distribution and number of the included studies are depicted in Figure 2. Eight of the studies collected information about the specific valve lesions of patients, two of which were studies of pregnant women with rheumatic MS (33,47). We found variations in the classification of MS across studies. MS was reported in 357 participants across seven studies; severe MS was reported in 116. In one of these studies, approximately half of participants had an MVA of less than 1.3 cm^2^ and 29% had an MVA of ≤1 cm (33). Joint guidelines from the American Society of Echocardiography and the European Association of Echocardiography classify moderate MS as MVA between 1 and 1.5 cm^2^ and severe MS as MVA < 1 cm^2^ (48). In the other study, 55.2% (n = 32) of participants had severe MS, defined as MVA ≤ 1 cm^2^ (47). One study did not give specific measurements but stated that 27.5% of participants had mitral regurgitation and 20.8% had MS (41). Among the other five studies, there were a total of 327 participants with RHD, of which mitral valve lesions were predominant. Aortic lesions were much less common; aortic stenosis was reported in 14 participants, and AR was reported in 49 participants across these studies.

Cardiac complications of pregnant women with RHD in the included studies are shown in Table 2. No participants had a known diagnosis of acute coronary syndrome. Only one valvular complication was captured, a thrombosed mechanical valve (32). Heart failure was the most common cardiac complication across all included studies, occurring in 12.9% to 100% of study participants. Arrhythmia was the second most common cardiac complication, reported in eight studies, ranging from 1.1% to 36% of study participants. One study did not have any deaths in their sample, whereas the remaining eight studies reported deaths due to cardiac causes (median: six deaths due to cardiac disease; total number of deaths: 56). Infective endocarditis and stroke were uncommon, each occurring in a total of three study participants.

**Table 2:** Qualitative summary of cardiac complications in pregnant women with cardiac disease, largely RHD.


AUTHOR (YEAR)	SAMPLE WITH CARDIAC DISEASE	SAMPLE WITH RHD	ANY CARDIAC COMPLICATION, n (%)	HEART FAILURE, n (%)	ARRHYTHMIA, n (%)	IE, n (%)	STROKE, n (%)	ARTERIAL OR VTE, n (%)	DEATH DUE TO CARDIAC CAUSE, n (%)

**Beaton et al. (2019)**	58	51	51.8%*	33%*	3.6%*	N/A	N/A	N/A	1 (1.7%)

**Desai et al. (2000)**	128	128	Not clearly stated	N/A	2 (1.6%)	1 (0.8%)	N/A	1 (0.8%)	0

**Diao et al. (2011)**	50	46	Not clearly stated	24 (48%)	18 (36%)	N/A	N/A	2 (4%)	13 (26%)

**Gebremedhin et al. (2022)**	58	58	Not clearly stated	40 (68.9%)	15 (25.9%)	N/A	N/A	2 (3.4%)	N/A

**Hailu et al. (2019)**	21	20	20 (95.2%)	20 (95.2%)	N/A	N/A	N/A	1 (4.8%)	3 (14.3%)

**Lumsden et al. (2020)**	97	73	54 (55.7%)	41 (42.2%)	7 (7.2%)	N/A	2 (2.1%)	10 (10.3%)	6 (6.2%)

**Nqayana et al. (2008)**	95	77	30 (31.5%)	10**(12.9%)	11**(14.3%)	2** (2.6%)	1**(1.3%)	N/A	13 (13.7%)

**Poli et al. (2020)**	91	80	54 (59.3%)	21 (23.1%)	14 (15.4%)	N/A	N/A	N/A	8 (8.8%)

**Soma-Pillay et al. (2008)**	189	120	Not clearly stated	N/A	2 (1.1%)	N/A	N/A	N/A	4 (2.1%)


Percentages given out of the total sample with cardiac disease. ACS: acute coronary syndrome; IE: infective endocarditis; N/A: not available; RHD: rheumatic heart disease; VTE: venous thromboembolism. *Provided prevalence/100 **Specific to participants with RHD.

Obstetric complications of pregnant women with RHD in the included studies are shown in Table 3. Pregnancy loss (encompassing early pregnancy loss and intrauterine fetal demise (IUFD) or stillbirth) was recorded in all nine studies, ranging from 5.5 to 22.4% of participants. In one study, early pregnancy losses made up most pregnancy losses (47); in several other studies, IUFD/stillbirth comprised more of the cases (33,34,37). Neonatal death was recorded in four of the studies, occurring in <5%. Preterm delivery was common and captured in all the included studies, ranging from 5.2% to 35.2%. Though low birth weight had variable definitions across studies, it was reported in four studies; up to one-quarter of neonates were born at low birth weight. Maternal death occurred in 44 individuals across seven studies. Maternal death rates, most of them due to cardiac disease, ranged from 1.7% to 34% in our studies. Seventeen individuals died in one study, representing 34% of the study participants (38).

**Table 3:** Qualitative summary of obstetric complications in pregnant women with cardiac disease, largely RHD.


AUTHOR (YEAR)	SAMPLE WITH CARDIAC DISEASE	SAMPLE WITH RHD	ANY OBSTETRIC COMPLICATION, n (%)	ANY PREGNANCY LOSS, n (%)	EARLY PREGNANCY LOSS, n (%)	IUFD OR STILLBIRTH, n (%)	NEWBORN (NEONATAL) DEATH, n (%)	PRE-TERM DELIVERY, n (%)	IUGR OR LOW BIRTH WEIGHT, n (%)	PREECLAMPSIA, n (%)	PPH, n (%)	MATERNAL DEATH, n (%)

**Beaton et al. (2019)**	58	51	Not clearly stated	7.1%*	N/A	4 (6.9%)	3 (5.2%)	3 (5.2%)	N/A	N/A	N/A	1 (1.7%)

**Desai et al. (2000)**	128	128	Not clearly stated	14 (10.9%)	6 (4.7%)	8 (6.3%)	N/A	45 (35.2%)	20 (16.7% out of 120)	10 (7.8%)	N/A	0

**Diao et al. (2011)**	50	46	Not clearly stated	4 (8%)	N/A	4 (8%)	Not clearly stated	5 (10%)	N/A	N/A	N/A	17 (34%)

**Gebremedhin et al. (2022)**	58	58	Not clearly stated	13 (22.4%)	10 (17.2%)	3 (5.2%)	N/A	8 (13.8%)	13 (22.4%)	N/A	N/A	4 (6.9%)

**Hailu et al. (2019)**	21	20	Not clearly stated	2 (9.5%)	Not clearly stated	2 (9.5%)	1 (4.8%)	3 (14.3%)	N/A	5 (23.8%)	N/A	3 (14.3%)

**Lumsden et al. (2020)**	97	73	63 (64.9%)	10 (10.3%)	N/A	10 (10.3%)	4 (4.1%)	32 (33%)	24 (24.7%)	14 (14.4%)	5 (5.2%)	6 (6.2%)

**Nqayana et al. (2008)**	95	77	Not clearly stated	6(7.8%)**	1(1.3%)**	5(6.5%)**	1(1.3%)**	14(18.2%)**	10(13%)**	1(1.3%)**	4(5.2%)**	N/A

**Poli et al. (2020)**	91	80	68 (74.7%)	5 (5.5%)	3 (3.3%)	2 (2.2%)	N/A	20 (22%)	N/A	13 (14.2%)	10 (10.9%)	8 (8.8%)

**Soma-Pillay et al. (2008)**	189	120	Not clearly stated	22 (11.6%)	N/A	N/A	N/A	18 (9.5%) delivered before 34w	N/A	N/A	N/A	5 (2.6%)


Percentages given out of the total sample with cardiac disease. *Provided prevalence/1000 **Specific to participants with RHD. IUFD: intrauterine fetal demise; IUGR: intra-uterine growth restriction; N/A: not available; PPH: post-partum hemorrhage; RHD: rheumatic heart disease.

A total of 28 patients from two studies received mitral valvulotomy during pregnancy for MS, and one patient received a mitral valve replacement at 32 weeks gestation (32,33). The remaining studies did not report on interventions for RHD in pregnancy.

### Quality assessment

Full assessments of risk of bias according to JBI are presented in Supplemental Tables 3–5 for case series, cohort, and case-control studies, respectively. Many studies were case series assessing outcomes of pregnant women with cardiac disease; therefore, we were cautious in interpreting results lacking control groups and using mostly descriptive analysis. Bias in the measurement of outcomes may be introduced in some of the case series; several did not use clear definitions of outcomes in the methods section. The case-control study and cohort study had stronger definitions and adjusted for confounding variables. Overall, studies maintained a level of quality sufficient for inclusion in this systematic review.

## Discussion

Our systematic review addressed gaps in the literature regarding the significant morbidity and mortality that arises during pregnancy for women with RHD and their neonates, especially in LMICs. We focused on SSA in this review as an area facing a disproportionate burden of RHD and its complications. Our review determined that heart failure and arrhythmias are major cardiac complications of RHD in pregnant women in SSA. Maternal mortality ranged from 1.7% to 34%, and death due to cardiac disease occurred in 7% of all pregnant women with RHD in this review. We found that pregnancy loss, preterm delivery, and intrauterine growth restriction are key contributors to maternal and fetal morbidity. Though severe MS was a common valvular lesion in this population, few patients across our included studies received mitral valvulotomy during pregnancy, and there was a singular mitral valve replacement reported during pregnancy.

Previous summative reviews on cardiac and obstetric outcomes of women with RHD have not focused on SSA, or reviews in the region have not been focused on one or more of our outcomes of interest (49,50). This may be due to the lack of studies published on this topic, as our search results suggest, or attributed to limited research capacity and lack of electronic medical record keeping. SSA encompasses 49 countries of various sizes, governments, environments, and healthcare settings. Notably, our nine included studies come from an upper-middle-income country (South Africa), two lower-middle-income countries (Kenya, Senegal), and two low-income countries (Ethiopia, Uganda).

### Cardiac complications

Heart failure was also the most common cause of hospital admission in pregnant women with rheumatic mitral valve disease in the ROPAC study, a large multicenter prospective cohort study located in countries with emerging economies, including South Africa. ROPAC reported combined outcomes for emerging countries without country-specific data and was therefore not included in our formal analysis. Heart failure occurred in 26.7% of the ROPAC cohort, which is the lower end of reported heart failure in our nine studies (16). In a Brazilian cohort of pregnant women with RHD, 21.4% experienced a cardiac complication, of which the majority (71.4%) had heart failure or pulmonary congestion, 16.8% had arrhythmia, and 1.7% had infective endocarditis (51). Heart failure/pulmonary congestion only occurred in 15.3% of the total sample with RHD, in contrast to our studies, in which the incidence of heart failure trended higher. In a Canadian cohort of women with rheumatic MS, 35% of pregnancies were associated with maternal cardiac complications, which were significantly higher in women with severe MS compared to moderate and mild MS, and no maternal deaths occurred (52). Predictors of cardiac complications included moderate or severe MS, pulmonary hypertension, and a history of valve repair (ibid.). These global trends are consistent with the higher rates of cardiac complications we observed in our studies. Overall, maternal mortality ranged from 1.7% to 34% in our studies; a systematic review focused on South Asia found a pooled maternal mortality rate of 26.1 per 1,000 women with cardiac disease (49).

### Obstetric complications

Obstetric complications were frequently observed across the reviewed studies, though not comprehensively captured in most studies. Rates of obstetric (8%) and fetal events (30%) were lower in the study of Canadian women than in our qualitative estimates (52). Among these, preterm labor/delivery was the most frequently reported obstetric event in our review, with incidence ranging from 5.2% to 35.2%. This aligns with an international systematic review and meta-analysis (with two included studies from South Africa) in which preterm birth was the most reported obstetric adverse event in pregnancies with RHD (50). Hameed et al., in a study from the US of a majority Hispanic population, found a statistically significant decrease in the duration of pregnancy, an increase in the incidence of premature deliveries, and an increase in the incidence of IUGR among patients with valve disease (53).

Spontaneous pregnancy loss was another recurring outcome reported in all our included studies (incidence ranged from 5.5% to 22.4%). The highest incidence of IUFD or stillbirth was 10.3%. Neonatal death was less common or less often reported. The ROPAC study noted miscarriage or fetal death in 5.1% of women with MS and 3.4% of women with MR (16).

IUGR, or low birth weight, was a common problem occurring in neonates of mothers with RHD, occurring in up to one-quarter of infants in our studies, which is similar to estimates from multiple studies performed in India (54). Prior surgical correction of valvular lesions may protect against lower neonatal birth weights (55). There is not a pathological link between preeclampsia and RHD that has been directly studied, though it is known that low-income individuals are at higher risk and rates are higher in women with rheumatic diseases such as systemic lupus erythematosus (56,57). We noted preeclampsia in 1.3–23.8% of the pregnant women in our studies. This observation should be further elucidated to determine if there is a link between RHD and preeclampsia. Pregnancies impacted by RHD are associated with a higher risk of adverse outcomes for both mother and fetus in LMICs compared to high-income nations and in SSA particularly.

### Valve interventions

Though pregnant women in SSA have high rates of severe disease, particularly MS, and are at high risk for complications, valve interventions during pregnancy are rarely performed. The low (n = 29) number of patients receiving valve interventions during pregnancy in our included studies is consistent with findings from other studies and registries in LMICs. The VALVAFRIC study, a multicenter registry of over 3,000 patients with RHD in eight Western and Central African countries that characterizes echocardiographic features, symptoms, interventions, and sociodemographic characteristics, reported that only 2.2% of patients qualifying for surgical intervention were operated on; all of these patients received surgical care abroad (58). In the ROPAC registry, less than 5% of patients received an intervention during pregnancy (14 balloon mitral commissurotomy and two surgical mitral valve replacements) (16). A retrospective study in India reported that 29 women received balloon mitral valvotomy during pregnancy (10.6%) (54).

If valve replacement surgery is pursued, the type of valve implanted—mechanical or bioprosthetic—has lasting implications. Mechanical valve replacement requires lifelong anticoagulation with a vitamin K antagonist, typically warfarin (20). Warfarin is a known teratogen; levels may be decreased during pregnancy or alternative anticoagulants may be offered that are associated with higher maternal complications (20). Bioprosthetic valves do not require lifelong anticoagulation; however, in young patients, the risk for degeneration of the valve is high.

In resource-limited settings, the optimal management for advanced RHD in pregnancy is unclear, with each pathway posing risks. Severe native valve dysfunction during pregnancy, especially with heart failure or pulmonary hypertension, increases the odds for poor outcomes for mother and baby; however, valve intervention, cardiopulmonary bypass, and postoperative management must be navigated with caution.

### Implications and areas for future research

Understanding the complications of RHD in pregnant women is key to counseling patients of reproductive age. Indeed, we know that current efforts to encourage safe pregnancies in women with RHD in SSA face challenges such as affordability and cultural acceptance of contraception, underdiagnosis of RHD, and patient education surrounding the disease. Raising public awareness and championing public health efforts in RHD screening are key tools for addressing the burden of RHD. Routine antenatal echocardiographic screening for RHD presents an important strategy in identifying previously undiagnosed RHD early in pregnancy (59,60). Efforts to bring diagnostic echocardiography to high-risk populations have surged in recent years, a crucial step forward to diagnose RHD in pediatric populations (61,62,63,64).

One Tanzanian study at a tertiary cardiac center found that the proportion of reproductive-age women with RHD using contraception was 7.1%, though the majority of participants in the study were high-risk according to the modified WHO maternal risk classification system for predicting adverse pregnancy outcomes among women with cardiac disease (65). A qualitative study from Uganda of postpartum women with RHD highlighted the central role of the spouse in medical decision-making (66). Educational programs for women with RHD are indispensable; future efforts may focus on support groups or patient-led initiatives to provide resources and teaching that can empower reproductive health. These efforts may involve the partners and spouses of women with RHD to facilitate shared understanding (67).

Another key component to enhance clinical care of women with RHD is implementing prospective registries to track contemporary outcomes of women during pregnancy. The VALVAFRIC and ROPAC studies are models for cohort studies (retrospective and prospective) that can be translated to future international efforts (58). Moi Teaching and Referral Hospital (MTRH) in Eldoret, Kenya, is aiming to address this gap by creating a prospective structural heart disease registry that is inclusive of obstetric history and complications. Future research can leverage the foundations of such registries for large-scale initiatives bridging multiple centers.

The 2015 Addis Ababa roadmap of actions to eradicate RHD in Africa recommends expanding access to reproductive health services for women with RHD and establishing prospective RHD registries (68). Another key component of their recommendations is establishing cardiac surgery centers of excellence. Valve repairs and replacements are uncommon in SSA due to a paucity of resources, though it is estimated that the need for cardiac surgery to address RHD in SSA is 200–250 per million (22,23,24). Suggestions for surgical capacity building in SSA include mentorship models between HICs and LMICs, outcome tracking, and value-based care (69). Such efforts require strong government backing and medical insurance systems that will support surgical interventions (70).

Risk calculators for pregnancies in patients with cardiovascular disease that are commonly utilized in high-income countries, such as the Cardiac Disease in Pregnancy Study (CARPREG II) and modified WHO prediction tools, do not accurately predict risk outside of high-income countries (37,71). The growing field of cardio-obstetrics offers a pathway to patients receiving specialty care for RHD in pregnancy, though has yet to be formally established in many settings. Joint cardio-obstetrics clinics offer the opportunity to validate and integrate pregnancy risk classifications into routine RHD care and serve as a medical home for the management of RHD in pregnant women.

### Strengths and Limitations

The main strengths of our review are the focus on SSA, consideration of both cardiac and obstetrics outcomes, and adherence to standard review protocols. There are also limitations to consider. This review should not be taken to summarize the issue of RHD in pregnancy for much of a continent but instead offers a high-level overview of the published literature on complications that pregnant women with RHD and their neonates may encounter in a vast region that faces historical and current social, economic, and political challenges. Definitions on obstetric and cardiac complications in pregnant women with RHD were not collected systematically across studies, though we applied standardized definitions in our protocol. Many articles presented combined clinical data for patients with RHD and patients with other cardiac conditions; therefore, our review of the impact of RHD on outcomes was less precise from those studies. We attempted to mitigate this limitation by only including studies in which most participants had RHD. Most included studies were performed in tertiary referral hospitals with small sample sizes of individuals with cardiac disease. We did not conduct a meta-analysis or assess statistical effect heterogeneity; therefore, we cannot assume precision of our estimates. We chose to focus mainly on maternal outcomes within pregnancy and the immediate postpartum period; future studies are needed to assess the consequences of RHD on the children of affected mothers and follow fetal outcomes into childhood.

## Conclusions

Pregnant women with RHD in SSA often have severe disease and are at risk for both cardiac and obstetric outcomes, particularly heart failure and preterm labor. Rates of these outcomes tended to be higher than those in international counterparts, while valve intervention rates were lower. Our findings provide support for future research and interventions, such as registries focused on pregnant women with RHD and educational programs for women of reproductive age with RHD. Future efforts should evaluate the feasibility of scaling cardiac surgery and interventions. Focus should be given to preconception counseling and strengthening of cardio-obstetrics specialty care for affected women who choose to conceive for improved patient outcomes.

## Additional File

The additional file for this article can be found as follows:

10.5334/gh.1522.s1 Supplementary material. Supplementary Tables 1 to 5.

## References

[1] World Health Organization. Rheumatic heart disease [Internet]. World Health Organization [cited 2018 May 8]. Available from: https://www.who.int/news-room/fact-sheets/detail/rheumatic-heart-disease

[2] Carapetis JR, Steer AC, Mulholland EK, Weber M. The global burden of group A streptococcal diseases. The Lancet Infectious Diseases. 2005;5(11):685–694. DOI: 10.1016/S1473-3099(05)70267-X 16253886

[3] Yuyun MF, Sliwa K, Kengne AP, Mocumbi AO, Bukhman G. Cardiovascular diseases in sub-Saharan Africa compared to high-income countries: An epidemiological perspective. 2020;15(1):15. DOI: 10.5334/gh.403 PMC7218780 32489788

[4] Woldu B, Bloomfield GS. Rheumatic heart disease in the twenty-first century. Current Cardiology Reports. 2016;18(10):96. DOI: 10.1007/s11886-016-0773-2 27566329 PMC9067597

[5] Watkins DA, Johnson CO, Colquhoun SM, et al. Global, regional, and national burden of rheumatic heart disease, 1990–2015. New England Journal of Medicine. 2017;377(8):713–722. DOI: 10.1056/NEJMoa1603693 28834488

[6] Roth GA, Mensah GA, Johnson CO, et al. Global burden of cardiovascular diseases and risk factors, 1990–2019. Journal of the American College of Cardiology. 2020;76(25):2982–3021. DOI: 10.1016/j.jacc.2020.11.010 33309175 PMC7755038

[7] Ghamari S, Abbasi-Kangevari M, Saeedi Moghaddam S, et al. Rheumatic heart disease is a neglected disease relative to its burden worldwide: Findings from global burden of disease 2019. Journal of the American Heart Association. 2022;11(13):e025284. DOI: 10.1161/JAHA.122.025284 35730651 PMC9333364

[8] Zühlke L, Engel ME, Karthikeyan G, et al. Characteristics, complications, and gaps in evidence-based interventions in rheumatic heart disease: The Global Rheumatic Heart Disease Registry (the REMEDY study). European Heart Journal. 2015;36(18):1115–1122. DOI: 10.1093/eurheartj/ehu449 25425448 PMC4422972

[9] Lumsden RH, Akwanalo C, Chepkwony S, et al. Clinical and geographic patterns of rheumatic heart disease in outpatients attending cardiology clinic in western Kenya. International Journal of Cardiology. 2016;223:228–235. DOI: 10.1016/j.ijcard.2016.08.069 27541662

[10] Eberly LA, Rusingiza E, Park PH, et al. Understanding the etiology of Heart Failure among the rural poor in sub-Saharan Africa: A 10-year experience from district hospitals in Rwanda. Journal of Cardiac Failure. 2018;24(12):849–853. DOI: 10.1016/j.cardfail.2018.10.002 30312764

[11] Sliwa K, Carrington M, Mayosi BM, Zigiriadis E, Mvungi R, Stewart S. Incidence and characteristics of newly diagnosed rheumatic heart disease in Urban African adults: insights from the Heart of Soweto Study. European Heart Journal. 2010;31(6):719–727. DOI: 10.1093/eurheartj/ehp530 19995873

[12] Goldstein SA, Ward CC. Congenital and acquired valvular heart disease in pregnancy. Current Cardiology Reports. 2017;19(10):96. DOI: 10.1007/s11886-017-0910-6 28840470

[13] Lewey J, Andrade L, Levine LD. Valvular Heart Disease in Pregnancy. Cardiology Clinics. 2021;39(1):151–161. ISSN 0733-8651, ISBN 9780323809269. DOI: 10.1016/j.ccl.2020.09.010 (https://www.sciencedirect.com/science/article/pii/S0733865120300874). 33222810 PMC8340680

[14] Carapetis JR, Beaton A, Cunningham MW, et al. Acute rheumatic fever and rheumatic heart disease. Nature Reviews Disease Primers. 2016;2(1):1–24. DOI: 10.1038/nrdp.2015.84 PMC5810582 27188830

[15] Anthony J, Osman A, Sani M. Valvular heart disease in pregnancy. Cardiovascular Journal of Africa. 2016;27(2):111–118. DOI: 10.5830/CVJA-2016-052 27213859 PMC4928166

[16] van Hagen IM, Thorne SA, Taha N, et al. Pregnancy outcomes in women with rheumatic mitral valve disease. Circulation. 2018;137(8):806–816. DOI: 10.1161/CIRCULATIONAHA.117.032561 29459466

[17] Orwat S, Diller GP, van Hagen IM, et al. Risk of pregnancy in moderate and severe aortic stenosis. Journal of the American College of Cardiology. 2016;68(16):1727–1737. DOI: 10.1016/j.jacc.2016.07.750 27737738

[18] Liaw J, Walker B, Hall L, Gorton S, White AV, Heal C. Rheumatic heart disease in pregnancy and neonatal outcomes: A systematic review and meta-analysis. PLOS ONE. 2021;16(6):e0253581. DOI: 10.1371/journal.pone.0253581 34185797 PMC8241043

[19] de Souza JAM, Martinez EE, Ambrose JA, et al. Percutaneous balloon mitral valvuloplasty in comparison with open mitral valve commissurotomy for mitral stenosis during pregnancy. Journal of the American College of Cardiology. 2001;37(3):900–903. DOI: 10.1016/S0735-1097(00)01184-0 11693768

[20] Otto CM, Nishimura RA, Bonow RO, et al. 2020 ACC/AHA guideline for the management of patients with valvular heart disease: A report of the American College of Cardiology/American Heart Association joint committee on clinical practice guidelines. Circulation. 2021;143(5):e72–e227. DOI: 10.1161/CIR.0000000000000923 33332150

[21] Kumar RK, Antunes MJ, Beaton A, et al. Contemporary diagnosis and management of rheumatic heart disease: Implications for closing the gap: A scientific statement from the American Heart Association. Circulation. 2020;142(20):e337–e357. DOI: 10.1161/CIR.0000000000000921 33073615

[22] Vervoort D, Antunes MJ, Pezzella AT. Rheumatic heart disease: The role of global cardiac surgery. Journal of Cardiac Surgery. 2021;36(8):2857–2864. DOI: 10.1111/jocs.15597 33938579

[23] Yankah C, Fynn-Thompson F, Antunes M, et al. Cardiac surgery capacity in sub-Saharan Africa: Quo vadis? The Thoracic and Cardiovascular Surgeon. 2014;62(05):393–401. DOI: 10.1055/s-0034-1383723 24955755

[24] Zilla P, Yacoub M, Zühlke L, et al. Global unmet needs in cardiac surgery. Global Heart. 2018;13(4):293–303. DOI: 10.1016/j.gheart.2018.08.002 30245177

[25] Kurjak A, Stanojević M, Dudenhausen J. Why maternal mortality in the world remains tragedy in low-income countries and shame for high-income ones: Will sustainable development goals (SDG) help? Journal of Perinatal Medicine. 2023;51(2):170–181. DOI: 10.1515/jpm-2022-0061 35636412

[26] Say L, Chou D, Gemmill A, et al. Global causes of maternal death: A WHO systematic analysis. The Lancet Global Health. 2014;2(6):e323–e333. DOI: 10.1016/S2214-109X(14)70227-X 25103301

[27] Soma-Pillay P, Seabe J, Soma-Pillay P, Seabe J, Sliwa K. The importance of cardiovascular pathology contributing to maternal death: Confidential enquiry into maternal deaths in South Africa, 2011–2013. Cardiovascular Journal of Africa. 2016;27(2):60. DOI: 10.5830/CVJA-2016-008 26895406 PMC4928161

[28] Page MJ, Moher D, Bossuyt PM, et al. PRISMA 2020 explanation and elaboration: Updated guidance and exemplars for reporting systematic reviews. BMJ. 2021;372:n160. DOI: 10.1136/bmj.n160 33781993 PMC8005925

[29] World Health Organizaion. Indicator metadata registry details [Internet]. World Health Organization [cited 2025 May 16]. Available from: https://www.who.int/data/gho/indicator-metadata-registry/imr-details/4622

[30] Moola S, Munn Z, Tufanaru C, Aromataris E, Sears K, Sfetcu R, et al. Systematic reviews of etiology and risk. In: Aromataris E & Munn Z (Eds.) JBI manual for evidence synthesis. JBI; 2020. pp. 256–261. Available from: https://synthesismanual.jbi.global. DOI: 10.46658/JBIRM-17-06

[31] Munn Z, Barker T, Moola S, Tufanaru C, Stern C, McArthur A, Stephenson M, et al. Methodological quality of case series studies. JBI Evidence Synthesis. 2020;18(10):2127–2133. DOI: 10.11124/JBISRIR-D-19-00099 33038125

[32] Nqayana T, Moodley J, Naidoo D. Cardiac disease in pregnancy. Cardiovascular Journal of Africa. 2008;19(3):145–151. https://journals.co.za/doi/pdf/10.10520/EJC23125 18568175 PMC3974559

[33] Desai DK, Adanlawo M, Naidoo DP, Moodley J, Kleinschmidt I. Mitral stenosis in pregnancy: A four-year experience at King Edward VIII Hospital, Durban, South Africa. British Journal of Obstetrics and Gynaecology. 2000;107:953–958. DOI: 10.1111/j.1471-0528.2000.tb10395.x 10955424

[34] Beaton A, Okello E, Scheel A, et al. Impact of heart disease on maternal, fetal and neonatal outcomes in a low-resource setting. Heart (British Cardiac Society). 2018;105(10):755. DOI: 10.1136/heartjnl-2018-313810 30415203 PMC11181686

[35] Roos-Hesselink J, Baris L, Johnson M, et al. Pregnancy outcomes in women with cardiovascular disease: Evolving trends over 10 years in the ESC Registry Of Pregnancy And Cardiac disease (ROPAC). European Heart Journal. 2019;40(47):3848–3855. DOI: 10.1093/eurheartj/ehz136 30907409

[36] Ferreira VV, Monteiro AV, Moreira RI, et al. Outcomes in pregnant women with valvular heart disease from Portuguese-speaking African countries treated in Portugal through an international agreement of health cooperation. Global Heart. 2023;18(1):4. DOI: 10.5334/gh.1183 36817227 PMC9936910

[37] Lumsden R, Barasa F, Park LP, et al. High burden of cardiac disease in pregnancy at a National Referral Hospital in western Kenya. Global Heart. 2020;15(1):10. DOI: 10.5334/gh.404 32489783 PMC7218778

[38] Diao M. Pregnancy in women with heart disease in sub-Saharan Africa. Archives of Cardiovascular Disease. 2011;104:370–374. DOI: 10.1016/j.acvd.2011.04.001 21798468

[39] Hailu A, Yeman A, Abate E, Teka H, Berhane H, Whe A. Management and outcome of severe pulmonary hypertension in pregnancy: Experience from a university hospital in northern Ethiopia. Ethiopian Medical Journal. 2019;57(2):203–210. https://scispace.com/pdf/management-and-outcome-of-pulmonary-hypertension-in-2qg0ufqbbm.pdf

[40] Poli PA, Orang’o EO, Mwangi A, Barasa FA. Factors related to maternal adverse outcomes in pregnant women with cardiac disease in low-resource settings. European Cardiology Review. 2020;15(e68). DOI: 10.15420/ecr.2020.04 PMC7709001 33304394

[41] Soma-Pillay P, MacDonald AP, Mathivha TM, Bakker JL, Mackintosh MO. Cardiac disease in pregnancy: a 4-year audit at Pretoria Academic Hospital. South African Medical Journal. 2008;98(7):553–556. 18785398

[42] Sliwa K, Azibani F, Baard J, et al. Reducing late maternal death due to cardiovascular disease – A pragmatic pilot study. International Journal of Cardiology. 2018;272:70–76. DOI: 10.1016/j.ijcard.2018.07.140 30087040

[43] Makgato C, Baloyi S, Nondabula T. Profile of cardiac patients who delivered at Universitas Academic Hospital (UAH) in Bloemfontein South Africa: 2012–2017. Obstetrics and Gynaecology Forum 2020. 30(2):28–32. https://hdl.handle.net/10520/EJC-1d8e552394

[44] Heemelaar S, Petrus A, Knight M, van den Akker T. Maternal mortality due to cardiac disease in low- and middle-income countries. Tropical Medicine and International Health. 2020;25(6):673–686. DOI: 10.1111/tmi.13386 32133737 PMC7318167

[45] Greeff N, Langenegger E, Herbst P, Lombard C, Theron A. Disease spectrum and short-term outcomes of obstetric patients with cardiac disease admitted to an obstetric critical care unit in South Africa. Southern African Journal of Anaesthesia and Analgesia. 2024;30(5):a1236. DOI: 10.36303/SAJAA.3120

[46] Balieva I, Voors AA, Balieva I, et al. Pregnancy in women with cardiac disease: a one-year retrospective review of management and maternal and neonatal outcomes in a tertiary hospital in Johannesburg, South Africa. Cardiovascular Journal of Africa. 2021;32(6):301. DOI: 10.5830/CVJA-2020-062 33559676 PMC8756042

[47] Gebremedhin Y, Guteta S, Melese B, Bekele E. Predictors of maternal and fetal outcomes of pregnant women with mitral stenosis. Published online 2022. DOI: 10.1101/2022.08.04.22278407

[48] Baumgartner H, Hung J, Bermejo J, et al. Echocardiographic assessment of valve stenosis: EAE/ASE recommendations for clinical practice. Journal of the American Society of Echocardiography. 2009;22(1):1–23. DOI: 10.1016/j.echo.2008.11.029 19130998

[49] Shrestha P, Kuikel S, Bajracharya S, et al. Pregnancy with heart disease in South Asia: A systematic review and meta-analysis of prevalence and outcome. Annals of Medicine & Surgery. 2022;80:104293. DOI: 10.1016/j.amsu.2022.104293 36045771 PMC9422311

[50] Liaw J, Walker B, Hall L, Gorton S, White AV, Heal C. Rheumatic heart disease in pregnancy and neonatal outcomes: A systematic review and meta-analysis. PLoS ONE. 2021;16(6):e0253581. DOI: 10.1371/journal.pone.0253581 34185797 PMC8241043

[51] Avila WS, Rossi EG, Ramires JAF, et al. Pregnancy in patients with heart disease: Experience with 1,000 cases. Clinical Cardiology. 2003;26(3):135–142. DOI: 10.1002/clc.4960260308 12685620 PMC6654765

[52] Silversides CK, Colman JM, Sermer M, Siu SC. Cardiac risk in pregnant women with rheumatic mitral stenosis. The American Journal of Cardiology. 2003;91(11):1382–1385. DOI: 10.1016/S0002-9149(03)00339-4 12767443

[53] Hameed A, Karaalp IS, Tummala PP, et al. The effect of valvular heart disease on maternal and fetal outcome of pregnancy. Journal of the American College of Cardiology. 2001;37(3):893–899. DOI: 10.1016/S0735-1097(00)01198-0 11693767

[54] Suri V, Sikka P, Singla R, Aggarwal N, Chopra S, Vijayvergiya R. Factors affecting the outcome of pregnancy with rheumatic heart disease: An experience from low-middle income country. Journal of Obstetrics and Gynaecology. 2019;39(8):1087–1092. DOI: 10.1080/01443615.2019.1587595 31195863

[55] Sethuraman D, Ramachandran N, Noorjahan S, Kanna V. Maternal and fetal outcomes in rheumatic heart disease in pregnancy. International Journal of Research in Medical Sciences. 2014;2(4):1632. DOI: 10.5455/2320-6012.ijrms20141172

[56] The Americnan College of Obstetrics & Gynecologists. Preeclampsia and pregnancy [Internet]. The American College of Obstetrics & Gynecologists; 2021 [cited 2025 June 10]. Available from: https://www.acog.org/womens-health/infographics/preeclampsia-and-pregnancy

[57] Milne ME, Clowse ME, Zhao C, Goldstein BA, Eudy AM. Impact of preeclampsia on infant and maternal health among women with rheumatic diseases. Lupus. 2024;33(4):397–402. DOI: 10.1177/09612033241235870 38413920

[58] Kingué S, Ba SA, Balde D, et al. The VALVAFRIC study: A registry of rheumatic heart disease in Western and Central Africa. Archives of Cardiovascular Diseases. 2016;109(5):321–329. DOI: 10.1016/j.acvd.2015.12.004 26988837

[59] Seitler S, Ahmad M, Ahuja SAC, et al. Routine antenatal echocardiography in high-prevalence areas of rheumatic heart disease: A WHO-guideline systematic review. Global Heart. 2024;19(1):39. DOI: 10.5334/gh.1318 38681969 PMC11049603

[60] Justin Paul G, Anne Princy S, Jeemon P. Routine antenatal echocardiography in low- and Middle-Income Countries. JACC: Advances. 2024;3(12, Part 2):101334. DOI: 10.1016/j.jacadv.2024.101334 39817097 PMC11733812

[61] Chillo P, Mutagaywa R, Nkya D, et al. Sub-clinical rheumatic heart disease (RHD) detected by hand-held echocardiogram in children participating in a school-based RHD prevention program in Tanzania. BMC Cardiovascular Disorders. 2023;23(1):155. DOI: 10.1186/s12872-023-03186-y 36966309 PMC10040127

[62] Nkoke C, Lekoubou A, Dzudie A, et al. Echocardiographic pattern of rheumatic valvular disease in a contemporary sub-Saharan African pediatric population: An audit of a major cardiac ultrasound unit in Yaounde, Cameroon. BMC Pediatrics. 2016;16:43. DOI: 10.1186/s12887-016-0584-z 27000111 PMC4800770

[63] Sims Sanyahumbi A, Sable CA, Beaton A, et al. School and community screening shows Malawi, Africa, to have a high prevalence of latent rheumatic heart disease. Congenital Heart Disease. 2016;11(6):615–621. DOI: 10.1111/chd.12353 27029239

[64] Beaton A, Lu JC, Aliku T, et al. The utility of handheld echocardiography for early rheumatic heart disease diagnosis: A field study. European Heart Journal – Cardiovascular Imaging. 2015;16(5):475–482. DOI: 10.1093/ehjci/jeu296 25564396 PMC4542771

[65] Paulo DG, Mutagaywa R, Mayala H, Barongo A. Pregnancy risk and contraception among reproductive-age women with rheumatic heart disease attending care at a tertiary cardiac center in Tanzania: A hospital-based cross-sectional study. BMC Women’s Health. 2023;23:404. DOI: 10.1186/s12905-023-02332-0 37653369 PMC10468869

[66] Voleti S, Okello E, Murali M, et al. The personal and clinical impact of screen-detected maternal rheumatic heart disease in Uganda: A prospective follow up study. BMC Pregnancy and Childbirth. 2020;20(1):611. DOI: 10.1186/s12884-020-03189-z 33036571 PMC7547429

[67] Fletcher R, Forbes F, Dadi AF, et al. Effect of male partners’ involvement and support on reproductive, maternal and child health and well-being in East Africa: A scoping review. Health Science Reports. 2024;7(8):e2269. DOI: 10.1002/hsr2.2269 39086507 PMC11286546

[68] Watkins D, Zuhlke L, Engel M, et al. Seven key actions to eradicate rheumatic heart disease in Africa: The Addis Ababa communiqué. The Cardiovascular Journal of Africa. 2016;27(3):184–187. DOI: 10.5830/CVJA-2015-090 26815006 PMC5125265

[69] Vera Vaz Ferreira MD, Endale Tefera MD, Oketcho Micheal MD, et al. Cardiac Surgery in Sub-Saharan Africa: Anthills of the Savannah. JACC: Advances. 2025;4(1). DOI: 10.1016/j.jacadv.2024.101223

[70] Effiom VB, Michael AJ, Ahmed FK, et al. Cardiothoracic surgery training in Africa: History and developments. JTCVS Open. 2024;19:370–377. DOI: 10.1016/j.xjon.2024.03.005 39015453 PMC11247221

[71] van Hagen IM, Boersma E, Johnson MR, et al. Global cardiac risk assessment in the Registry Of Pregnancy And Cardiac disease: Results of a registry from the European Society of Cardiology. European Journal of Heart Failure. 2016;18(5):523–533. DOI: 10.1002/ejhf.501 27006109

