# Supplementary material for: Cardiac and Obstetric Complications of Pregnant Women with Rheumatic Heart Disease in Sub-Saharan Africa: A Systematic Review
Source: Glob Heart. 2026 Jan 30;21(1):5. doi: 10.5334/gh.1522 (PMC12857620; doi:10.5334/gh.1522)
Supplement: Supplementary material. — Supplementary Tables 1 to 5. [file gh-21-1-1522-s1.pdf]

Supplemental Tables and Figures

Cardiac and obstetric complications of pregnant women with rheumatic heart disease in sub-Saharan Africa: a systematic review

Evangelia Alexopoulos, BS, BA, Doreen Nakagaayi, MBChB, MMed, Elizabeth R. Blackwood, Felix Barasa, MD, MMed, Joan Kiyeng, MD, MMed, Wycliffe Kosgei, MD, MMed, G. Titus Ng'eno, MD, MSc-GH, Shanti Nulu, MD, MPH, Rebecca Lumsden, MD, Andrea Beaton, MD, Gerald S. Bloomfield, MD, MPH

Table of Contents

Supplemental Table 1. Complete Search Strategies......... 2

Supplemental Table 2. Reasons for exclusion 15

Supplemental Table 3. Risk of bias for case series using the JBI critical appraisal checklist . 16

Supplemental Table 4. Risk of bias for cohort studies using the JBI critical appraisal checklist 17

Supplemental Table 5. Risk of bias for case-control studies using the JBI critical appraisal checklist......... 18

Supplemental References......... 19

Supplemental Table 1. Complete Search Strategies

Librarian Searcher: Beth Blackwood, MSLS; Duke University Medical Center Library & Archives, Duke University School of Medicine

MEDLINE (via PubMed)
Search date: 12/16/2024

Concept	Strategy	Results
#1 Pregnancy Terms	"Pregnancy"[Mesh] OR Pregnancy[tiab] OR pregnancies[tiab] OR pregnant[tiab] OR gestating[tiab] OR gestational[tiab] OR gestated[tiab] OR gestates[tiab] OR gestate[tiab] OR gestation[tiab] OR maternal[tiab] OR maternity[tiab]	1,367,667
#2 Rheumatic Heart Disease	"Rheumatic Heart Disease"[Mesh] OR “Mitral Valve Stenosis”[Mesh] OR "Mitral Valve Insufficiency"[Mesh] OR “Aortic valve Stenosis”[Mesh] OR "Tricuspid Valve Stenosis"[Mesh] OR "Pulmonary Valve Stenosis"[Mesh] OR "Rheumatic Heart Disease"[tiab] OR “rheumatic heart diseases”[tiab] OR “Bouillaud Disease”[tiab] OR “Bouillaud Diseases”[tiab] OR “Bouillaud’s Disease”[tiab] OR “Bouillaud’s Diseases”[tiab] OR “Bouillauds Disease”[tiab] OR “Bouillauds Diseases”[tiab] OR “mitral valve stenosis”[tiab] OR “Mitral valve stenoses”[tiab] OR “mitral stenosis”[tiab] OR “mitral stenoses”[tiab] OR “mitral valve regurgitation”[tiab] OR “mitral regurgitation”[tiab] OR “aortic valve stenosis”[tiab] OR “aortic valve stenoses”[tiab] OR “aortic stenosis”[tiab] OR “aortic stenoses”[tiab] OR “aortic regurgitation”[tiab] OR "Tricuspid Valve Stenosis"[tiab] OR "Tricuspid Valve Stenoses"[tiab] OR “tricuspid stenosis”[tiab] OR “tricuspid stenoses”[tiab] OR “tricuspid regurgitation”[tiab] OR "Pulmonary Valve Stenosis"[tiab] OR "Pulmonary Valve Stenoses"[tiab] OR "Pulmonary Stenosis"[tiab] OR "Pulmonary Stenoses"[tiab] OR “pulmonary regurgitation”[tiab]	141,090
#3 LMIC Terms	"Developing Countries"[Mesh] OR "Afghanistan"[Mesh] OR "Bangladesh"[Mesh] OR "Benin"[Mesh] OR "Burkina Faso"[Mesh] OR "Burundi"[Mesh] OR "Cambodia"[Mesh] OR "Central African Republic"[Mesh] OR "Chad"[Mesh] OR "Comoros"[Mesh] OR "Democratic Republic of the Congo"[Mesh] OR "Eritrea"[Mesh] OR "Ethiopia"[Mesh] OR "Gambia"[Mesh] OR "Guinea"[Mesh] OR "Guinea-Bissau"[Mesh] OR "Haiti"[Mesh] OR "Kenya"[Mesh] OR "Democratic People's Republic of Korea"[Mesh] OR "Liberia"[Mesh] OR "Madagascar"[Mesh] OR "Malawi"[Mesh] OR "Mali"[Mesh] OR "Mozambique"[Mesh] OR "Myanmar"[Mesh] OR "Nepal"[Mesh] OR "Niger"[Mesh] OR "Rwanda"[Mesh] OR "Sierra Leone"[Mesh] OR "Somalia"[Mesh] OR "Tajikistan"[Mesh] OR "Tanzania"[Mesh] OR "Togo"[Mesh] OR "Uganda"[Mesh] OR "Zimbabwe"[Mesh] OR "Armenia"[Mesh] OR "Bhutan"[Mesh] OR "Bolivia"[Mesh] OR "Cameroon"[Mesh] OR "Cabo Verde"[Mesh] OR "Congo"[Mesh] OR "Cote d'Ivoire"[Mesh] OR "Djibouti"[Mesh] OR "Egypt"[Mesh] OR "El Salvador"[Mesh] OR "Georgia (Republic)"[Mesh] OR "Ghana"[Mesh] OR "Guatemala"[Mesh] OR "Guyana"[Mesh] OR	8,535,630

	"Honduras"[Mesh] OR "Indonesia"[Mesh] OR "India"[Mesh] OR "Kosovo"[Mesh] OR "Kyrgyzstan"[Mesh] OR "Laos"[Mesh] OR "Lesotho"[Mesh] OR "Mauritania"[Mesh] OR "Micronesia"[Mesh] OR "Moldova"[Mesh] OR "Mongolia"[Mesh] OR "Morocco"[Mesh] OR "Nicaragua"[Mesh] OR "Nigeria"[Mesh] OR "Pakistan"[Mesh] OR "Papua New Guinea"[Mesh] OR "Paraguay"[Mesh] OR "Philippines"[Mesh] OR "Independent State of Samoa"[Mesh] OR "Atlantic Islands"[Mesh] OR "Senegal"[Mesh] OR "Melanesia"[Mesh] OR "Sri Lanka"[Mesh] OR "Sudan"[Mesh] OR "Eswatini"[Mesh] OR "Syria"[Mesh] OR "Timor-Leste"[Mesh] OR "Ukraine"[Mesh] OR "Uzbekistan"[Mesh] OR "Vanuatu"[Mesh] OR "Vietnam"[Mesh] OR "Middle East"[Mesh] OR "Yemen"[Mesh] OR "Zambia"[Mesh] OR "Angola"[Mesh] OR "Albania"[Mesh] OR "Algeria"[Mesh] OR "American Samoa"[Mesh] OR "Argentina"[Mesh] OR "Azerbaijan"[Mesh] OR "Republic of Belarus"[Mesh] OR "Belize"[Mesh] OR "Bosnia and Herzegovina"[Mesh] OR "Botswana"[Mesh] OR "Brazil"[Mesh] OR "Bulgaria"[Mesh] OR "Chile"[Mesh] OR "China"[Mesh] OR "Colombia"[Mesh] OR "Costa Rica"[Mesh] OR "Croatia"[Mesh] OR "Cuba"[Mesh] OR "Czech Republic"[Mesh] OR "Dominica"[Mesh] OR "Dominican Republic"[Mesh] OR "Ecuador"[Mesh] OR "Estonia"[Mesh] OR "Equatorial Guinea"[Mesh] OR "Fiji"[Mesh] OR "Gabon"[Mesh] OR "Grenada"[Mesh] OR "Iran"[Mesh] OR "Iraq"[Mesh] OR "Jamaica"[Mesh] OR "Jordan"[Mesh] OR "Kazakhstan"[Mesh] OR "Latvia"[Mesh] OR "Lebanon"[Mesh] OR "Libya"[Mesh] OR "Lithuania"[Mesh] OR "Republic of North Macedonia"[Mesh] OR "Malaysia"[Mesh] OR "Indian Ocean Islands"[Mesh] OR "Mexico"[Mesh] OR "Montenegro"[Mesh] OR "Namibia"[Mesh] OR "Palau"[Mesh] OR "Panama"[Mesh] OR "Poland"[Mesh] OR "Peru"[Mesh] OR "Romania"[Mesh] OR "Russia"[Mesh] OR "Serbia"[Mesh] OR "Seychelles"[Mesh] OR "South Africa"[Mesh] OR "Saint Lucia"[Mesh] OR "Saint Vincent and the Grenadines"[Mesh] OR "Slovakia"[Mesh] OR "Suriname"[Mesh] OR "Thailand"[Mesh] OR "Tonga"[Mesh] OR "Tunisia"[Mesh] OR "Turkey"[Mesh] OR "Turkmenistan"[Mesh] OR "Venezuela"[Mesh] OR "Afghanistan"[all fields] OR "Bangladesh"[all fields] OR "Benin"[all fields] OR "Burkina Faso"[all fields] OR "Burundi"[all fields] OR "Cambodia"[all fields] OR "cabo verde"[all fields] OR "Central African Republic"[all fields] OR "Chad"[all fields] OR "Comoros"[all fields] OR "Democratic Republic of the Congo"[all fields] OR "Eritrea"[all fields] OR "Ethiopia"[all fields] OR "Gambia"[all fields] OR "Guinea"[all fields] OR "Guinea-Bissau"[all fields] OR "Haiti"[all fields] OR "Kenya"[all fields] OR "Democratic People's Republic of Korea"[all fields] OR "Liberia"[all fields] OR "Madagascar"[all fields] OR "Malawi"[all fields] OR "Mali"[all fields] OR "Mozambique"[all fields] OR "Myanmar"[all fields] OR "Nepal"[all fields] OR "Niger"[all fields] OR "Rwanda"[all fields] OR "Sierra Leone"[all fields] OR "Somalia"[all fields] OR "Tajikistan"[all fields] OR "Tanzania"[all fields] OR "Togo"[all fields] OR "Uganda"[all fields] OR "Zimbabwe"[all fields] OR "Armenia"[all fields] OR "Bhutan"[all fields] OR "Bolivia"[all fields] OR "Cameroon"[all fields] OR "Cabo	

	Verde"[all fields] OR "Congo"[all fields] OR "Cote d'Ivoire"[all fields] OR "Djibouti"[all fields] OR "Egypt"[all fields] OR "El Salvador"[all fields] OR "Georgia"[all fields] OR "Ghana"[all fields] OR "Guatemala"[all fields] OR "Guyana"[all fields] OR "Honduras"[all fields] OR "Indonesia"[all fields] OR "India"[all fields] OR “Kiribati”[all fields] OR "Kosovo"[all fields] OR "Kyrgyzstan"[all fields] OR "Kyrgyz"[all fields] OR "Laos"[all fields] OR "lao"[all fields] OR "Lesotho"[all fields] OR "Mauritania"[all fields] OR "Micronesia"[all fields] OR "mariana"[all fields] OR "Moldova"[all fields] OR "Mongolia"[all fields] OR "Morocco"[all fields] OR "Nicaragua"[all fields] OR "Nigeria"[all fields] OR "Pakistan"[all fields] OR "Papua New Guinea"[all fields] OR "Paraguay"[all fields] OR "Philippines"[all fields] OR "Poland"[all fields] OR "Independent State of Samoa"[all fields] OR "Atlantic Islands"[all fields] OR "Sao Tome"[all fields] OR Principe[all fields] OR "Senegal"[all fields] OR "Melanesia"[all fields] OR "Solomon islands"[all fields] OR "Sri Lanka"[all fields] OR "Sudan"[all fields] OR "Swaziland"[all fields] OR "Eswatini"[all fields] OR "Syria"[all fields] OR "East Timor"[all fields] OR "Timor leste"[all fields] OR "Ukraine"[all fields] OR "Uzbekistan"[all fields] OR "Vanuatu"[all fields] OR "Vietnam"[all fields] OR "Middle East"[all fields] OR "west bank"[all fields] OR "Gaza"[all fields] OR "Yemen"[all fields] OR "Zambia"[all fields] OR "Angola"[all fields] OR "Albania"[all fields] OR "Algeria"[all fields] OR "Argentina"[all fields] OR "Samoa"[all fields] OR "Azerbaijan"[all fields] OR "Republic of Belarus"[all fields] OR "Belize"[all fields] OR "Bosnia-Herzegovina"[all fields] OR "Botswana"[all fields] OR "Brazil"[all fields] OR "Bulgaria"[all fields] OR "Chile"[all fields] OR "China"[all fields] OR "Colombia"[all fields] OR "Costa Rica"[all fields] OR "Croatia"[all fields] OR "Cuba"[all fields] OR "Czech"[all fields] OR "Czechoslovakia"[all fields] OR "Dominica"[all fields] OR "Dominican Republic"[all fields] OR "Ecuador"[all fields] OR "Estonia"[all fields] OR "Equatorial Guinea"[all fields] OR "Fiji"[all fields] OR "Gabon"[all fields] OR "Grenada"[all fields] OR "Iran"[all fields] OR "Iraq"[all fields] OR "Jamaica"[all fields] OR "Jordan"[all fields] OR "Kazakhstan"[all fields] OR "Latvia"[all fields] OR "Lebanon"[all fields] OR "Libya"[all fields] OR "Lithuania"[all fields] OR "Macedonia"[all fields] OR "Malaysia"[all fields] OR "Indian Ocean Islands"[all fields] OR "Maldives"[all fields] OR “Marshall Islands”[all fields] OR "Mauritius"[all fields] OR "Mexico"[all fields] OR "Montenegro"[all fields] OR "Namibia"[all fields] OR "Palau"[all fields] OR "Panama"[all fields] OR "Peru"[all fields] OR "Romania"[all fields] OR "Russia"[all fields] OR "Russian Federation"[all fields] OR "Serbia"[all fields] OR "Seychelles"[all fields] OR "Slovakia"[all fields] OR "Slovak"[all fields] OR "South Africa"[all fields] OR "Saint Lucia"[all fields] OR "Saint Vincent and the Grenadines"[all fields] OR "Suriname"[all fields] OR "Thailand"[all fields] OR "Tonga"[all fields] OR "Tunisia"[all fields] OR "Turkey"[all fields] OR "Turkmenistan"[all fields] OR "Tuvalu"[all fields] OR "Venezuela"[all fields] OR Palestine[all fields] OR Palestinian[all fields] OR "low resource"[all fields] OR "under-resourced"[all fields] OR "resource	

	poor"[all fields] OR "under-developed"[all fields] OR "underdeveloped"[all fields] OR "developing country"[all fields] OR "developing countries"[all fields] OR "developing world"[all fields] OR "third world"[all fields] OR lmic[all fields] OR (low[all fields] AND middle[all fields] AND income[all fields])	
#4 Combined	#1 AND #2 AND #3	857
Validation String	30415203 OR 32489783 OR 33559676 OR 18785398 OR 35129101 OR 26895406 OR 27772597 OR 21798468	8/8

Embase (via Elsevier)
Search date: 12/16/2024

**Concept**	**Strategy**	**Results**
#1 Pregnancy Terms	'pregnancy'/exp OR (Pregnancy OR pregnancies OR pregnant OR gestating OR gestational OR gestated OR gestates OR gestate OR gestation OR maternal OR maternity):ti,ab	1,589,380
#2 Rheumatic Heart Disease	'rheumatic heart disease'/exp OR 'mitral valve stenosis'/exp OR 'aortic valve stenosis'/exp OR 'tricuspid valve stenosis'/exp OR 'pulmonary valve stenosis'/exp OR ("Rheumatic Heart Disease" OR “rheumatic heart diseases” OR “Bouillaud Disease” OR “Bouillaud Diseases” OR “Bouillauds Disease” OR “Bouillauds Diseases” OR “mitral valve stenosis” OR “Mitral valve stenoses” OR “mitral stenosis” OR “mitral stenoses” OR “mitral valve regurgitation” OR “mitral regurgitation” OR “aortic valve stenosis” OR “aortic valve stenoses” OR “aortic stenosis” OR “aortic stenoses” OR “aortic regurgitation” OR "Tricuspid Valve Stenosis" OR "Tricuspid Valve Stenoses" OR “tricuspid stenosis” OR “tricuspid stenoses” OR “tricuspid regurgitation” OR "Pulmonary Valve Stenosis" OR "Pulmonary Valve Stenoses" OR "Pulmonary Stenosis" OR "Pulmonary Stenoses" OR “pulmonary regurgitation”):ti,ab	162,143
#3 LMIC Terms	'Developing Countries'/exp OR 'Afghanistan'/exp OR 'Bangladesh'/exp OR 'Benin'/exp OR 'Burkina Faso'/exp OR 'Burundi'/exp OR 'Cambodia'/exp OR 'Central African Republic'/exp OR 'Chad'/exp OR 'Comoros'/exp OR 'Democratic Republic of the Congo'/exp OR 'Eritrea'/exp OR 'Ethiopia'/exp OR 'Gambia'/exp OR 'Guinea'/exp OR 'Guinea-Bissau'/exp OR 'Haiti'/exp OR 'Kenya'/exp OR 'Democratic Peoples Republic of Korea'/exp OR 'Liberia'/exp OR 'Madagascar'/exp OR 'Malawi'/exp OR 'Mali'/exp OR 'Mozambique'/exp OR 'Myanmar'/exp OR 'Nepal'/exp OR 'Niger'/exp OR 'Rwanda'/exp OR 'Sierra Leone'/exp OR 'Somalia'/exp OR 'Tajikistan'/exp OR 'Tanzania'/exp OR 'Togo'/exp OR 'Uganda'/exp OR 'Zimbabwe'/exp OR 'Armenia'/exp OR 'Bhutan'/exp OR 'Bolivia'/exp OR 'Cameroon'/exp OR 'Cabo Verde'/exp OR 'Congo'/exp OR 'Cote d Ivoire'/exp OR 'Djibouti'/exp OR 'Egypt'/exp OR 'El Salvador'/exp OR 'Georgia (Republic)'/exp OR 'Ghana'/exp OR 'Guatemala'/exp OR 'Guyana'/exp OR 'Honduras'/exp OR 'Indonesia'/exp OR 'India'/exp OR 'Kosovo'/exp OR 'Kyrgyzstan'/exp OR 'Laos'/exp OR 'Lesotho'/exp	2,891,213

	OR 'Mauritania'/exp OR 'Micronesia'/exp OR 'Moldova'/exp OR 'Mongolia'/exp OR 'Morocco'/exp OR 'Nicaragua'/exp OR 'Nigeria'/exp OR 'Pakistan'/exp OR 'Papua New Guinea'/exp OR 'Paraguay'/exp OR 'Philippines'/exp OR 'Independent State of Samoa'/exp OR 'Atlantic Islands'/exp OR 'Senegal'/exp OR 'Melanesia'/exp OR 'Sri Lanka'/exp OR 'Sudan'/exp OR 'Eswatini'/exp OR 'Syria'/exp OR 'Timor-Leste'/exp OR 'Ukraine'/exp OR 'Uzbekistan'/exp OR 'Vanuatu'/exp OR 'Vietnam'/exp OR 'Middle East'/exp OR 'Yemen'/exp OR 'Zambia'/exp OR 'Angola'/exp OR 'Albania'/exp OR 'Algeria'/exp OR 'American Samoa'/exp OR 'Argentina'/exp OR 'Azerbaijan'/exp OR 'Republic of Belarus'/exp OR 'Belize'/exp OR 'Bosnia and Herzegovina'/exp OR 'Botswana'/exp OR 'Brazil'/exp OR 'Bulgaria'/exp OR 'Chile'/exp OR 'China'/exp OR 'Colombia'/exp OR 'Costa Rica'/exp OR 'Croatia'/exp OR 'Cuba'/exp OR 'Czech Republic'/exp OR 'Dominica'/exp OR 'Dominican Republic'/exp OR 'Ecuador'/exp OR 'Estonia'/exp OR 'Equatorial Guinea'/exp OR 'Fiji'/exp OR 'Gabon'/exp OR 'Grenada'/exp OR 'Iran'/exp OR 'Iraq'/exp OR 'Jamaica'/exp OR 'Jordan'/exp OR 'Kazakhstan'/exp OR 'Latvia'/exp OR 'Lebanon'/exp OR 'Libya'/exp OR 'Lithuania'/exp OR 'Republic of North Macedonia'/exp OR 'Malaysia'/exp OR 'Indian Ocean Islands'/exp OR 'Mexico'/exp OR 'Montenegro'/exp OR 'Namibia'/exp OR 'Palau'/exp OR 'Panama'/exp OR 'Poland'/exp OR 'Peru'/exp OR 'Romania'/exp OR 'Russia'/exp OR 'Serbia'/exp OR 'Seychelles'/exp OR 'South Africa'/exp OR 'Saint Lucia'/exp OR 'Saint Vincent and the Grenadines'/exp OR 'Slovakia'/exp OR 'Suriname'/exp OR 'Thailand'/exp OR 'Tonga'/exp OR 'Tunisia'/exp OR 'Turkey'/exp OR 'Turkmenistan'/exp OR 'Venezuela'/exp OR ('Afghanistan' OR 'Bangladesh' OR 'Benin' OR 'Burkina Faso' OR 'Burundi' OR 'Cambodia' OR 'cabo verde' OR 'Central African Republic' OR 'Chad' OR 'Comoros' OR 'Democratic Republic of the Congo' OR 'Eritrea' OR 'Ethiopia' OR 'Gambia' OR 'Guinea' OR 'Guinea-Bissau' OR 'Haiti' OR 'Kenya' OR 'Democratic Peoples Republic of Korea' OR 'Liberia' OR 'Madagascar' OR 'Malawi' OR 'Mali' OR 'Mozambique' OR 'Myanmar' OR 'Nepal' OR 'Niger' OR 'Rwanda' OR 'Sierra Leone' OR 'Somalia' OR 'Tajikistan' OR 'Tanzania' OR 'Togo' OR 'Uganda' OR 'Zimbabwe' OR 'Armenia' OR 'Bhutan' OR 'Bolivia' OR 'Cameroon' OR 'Cabo Verde' OR 'Congo' OR 'Cote d Ivoire' OR 'Djibouti' OR 'Egypt' OR 'El Salvador' OR 'Georgia' OR 'Ghana' OR 'Guatemala' OR 'Guyana' OR 'Honduras' OR 'Indonesia' OR 'India' OR 'Kiribati' OR 'Kosovo' OR 'Kyrgyzstan' OR 'Kyrgyz' OR 'Laos' OR 'lao' OR 'Lesotho' OR 'Mauritania' OR 'Micronesia' OR 'mariana' OR 'Moldova' OR 'Mongolia' OR 'Morocco' OR 'Nicaragua' OR 'Nigeria' OR 'Pakistan' OR 'Papua New Guinea' OR 'Paraguay' OR 'Philippines' OR 'Poland' OR 'Independent State of Samoa' OR 'Atlantic Islands' OR 'Sao Tome' OR Principe OR 'Senegal' OR 'Melanesia' OR 'Solomon islands' OR 'Sri Lanka' OR 'Sudan' OR 'Swaziland' OR 'Eswatini' OR 'Syria' OR 'East Timor' OR 'Timor leste' OR 'Ukraine' OR 'Uzbekistan' OR 'Vanuatu' OR 'Vietnam' OR 'Middle East' OR 'west bank' OR 'Gaza' OR 'Yemen' OR 'Zambia' OR 'Angola' OR 'Albania' OR 'Algeria' OR 'Argentina' OR 'Samoa' OR 'Azerbaijan' OR 'Republic of Belarus' OR 'Belize' OR 'Bosnia-	

	Herzegovina' OR 'Botswana' OR 'Brazil' OR 'Bulgaria' OR 'Chile' OR 'China' OR 'Colombia' OR 'Costa Rica' OR 'Croatia' OR 'Cuba' OR 'Czech' OR 'Czechoslovakia' OR 'Dominica' OR 'Dominican Republic' OR 'Ecuador' OR 'Estonia' OR 'Equatorial Guinea' OR 'Fiji' OR 'Gabon' OR 'Grenada' OR 'Iran' OR 'Iraq' OR 'Jamaica' OR 'Jordan' OR 'Kazakhstan' OR 'Latvia' OR 'Lebanon' OR 'Libya' OR 'Lithuania' OR 'Macedonia' OR 'Malaysia' OR 'Indian Ocean Islands' OR 'Maldives' OR 'Marshall Islands' OR 'Mauritius' OR 'Mexico' OR 'Montenegro' OR 'Namibia' OR 'Palau' OR 'Panama' OR 'Peru' OR 'Romania' OR 'Russia' OR 'Russian Federation' OR 'Serbia' OR 'Seychelles' OR 'Slovakia' OR 'Slovak' OR 'South Africa' OR 'Saint Lucia' OR 'Saint Vincent and the Grenadines' OR 'Suriname' OR 'Thailand' OR 'Tonga' OR 'Tunisia' OR 'Turkey' OR 'Turkmenistan' OR 'Tuvalu' OR 'Venezuela' OR Palestine OR Palestinian OR 'low resource' OR 'under-resourced' OR 'resource poor' OR 'under-developed' OR 'underdeveloped' OR 'developing country' OR 'developing countries' OR 'developing world' OR 'third world' OR lmic OR (low AND middle AND income)):ti,ab	
#4 Combined	#1 AND #2 AND #3	636
#5	#4 NOT ('editorial'/exp OR [editorial]/lim OR 'note'/exp OR [note]/lim OR [conference abstract]/lim OR 'conference abstract'/exp)	445

Web of Science (via Clarivate)
Search date: 12/16/2024

Concept	Strategy	Results
#1 Pregnancy Terms	TS=(Pregnancy OR pregnancies OR pregnant OR gestating OR gestational OR gestated OR gestates OR gestate OR gestation OR maternal OR maternity)	1,037,334
#2 Rheumatic Heart Disease	TS=("Rheumatic Heart Disease" OR “rheumatic heart diseases” OR “Bouillaud Disease” OR “Bouillaud Diseases” OR “Bouillaud’s Disease” OR “Bouillaud’s Diseases” OR “Bouillauds Disease” OR “Bouillauds Diseases” OR “mitral valve stenosis” OR “Mitral valve stenoses” OR “mitral stenosis” OR “mitral stenoses” OR “mitral valve regurgitation” OR “mitral regurgitation” OR “aortic valve stenosis” OR “aortic valve stenoses” OR “aortic stenosis” OR “aortic stenoses” OR “aortic regurgitation” OR "Tricuspid Valve Stenosis" OR "Tricuspid Valve Stenoses" OR “tricuspid stenosis” OR “tricuspid stenoses” OR “tricuspid regurgitation” OR "Pulmonary Valve Stenosis" OR "Pulmonary Valve Stenoses" OR "Pulmonary Stenosis" OR "Pulmonary Stenoses" OR “pulmonary regurgitation”)	85,389
#3 LMIC Terms	TS=("Afghanistan" OR "Bangladesh" OR "Benin" OR "Burkina Faso" OR "Burundi" OR "Cambodia" OR "cabo verde" OR "Central African Republic" OR "Chad" OR "Comoros" OR "Democratic Republic of the Congo" OR "Eritrea" OR "Ethiopia" OR "Gambia" OR "Guinea" OR "Guinea-Bissau" OR "Haiti" OR "Kenya" OR "Democratic People's Republic of Korea" OR "Liberia" OR "Madagascar" OR "Malawi" OR "Mali" OR "Mozambique" OR "Myanmar" OR "Nepal"	4,915,467

	OR "Niger" OR "Rwanda" OR "Sierra Leone" OR "Somalia" OR "Tajikistan" OR "Tanzania" OR "Togo" OR "Uganda" OR "Zimbabwe" OR "Armenia" OR "Bhutan" OR "Bolivia" OR "Cameroon" OR "Cabo Verde" OR "Congo" OR "Cote d'Ivoire" OR "Djibouti" OR "Egypt" OR "El Salvador" OR "Georgia" OR "Ghana" OR "Guatemala" OR "Guyana" OR "Honduras" OR "Indonesia" OR "India" OR “Kiribati” OR "Kosovo" OR "Kyrgyzstan" OR "Kyrgyz" OR "Laos" OR "lao" OR "Lesotho" OR "Mauritania" OR "Micronesia" OR "mariana" OR "Moldova" OR "Mongolia" OR "Morocco" OR "Nicaragua" OR "Nigeria" OR "Pakistan" OR "Papua New Guinea" OR "Paraguay" OR "Philippines" OR "Poland" OR "Independent State of Samoa" OR "Atlantic Islands" OR "Sao Tome" OR Principe OR "Senegal" OR "Melanesia" OR "Solomon islands" OR "Sri Lanka" OR "Sudan" OR "Swaziland" OR "Eswatini" OR "Syria" OR "East Timor" OR "Timor leste" OR "Ukraine" OR "Uzbekistan" OR "Vanuatu" OR "Vietnam" OR "Middle East" OR "west bank" OR "Gaza" OR "Yemen" OR "Zambia" OR "Angola" OR "Albania" OR "Algeria" OR "Argentina" OR "Samoa" OR "Azerbaijan" OR "Republic of Belarus" OR "Belize" OR "Bosnia-Herzegovina" OR "Botswana" OR "Brazil" OR "Bulgaria" OR "Chile" OR "China" OR "Colombia" OR "Costa Rica" OR "Croatia" OR "Cuba" OR "Czech" OR "Czechoslovakia" OR "Dominica" OR "Dominican Republic" OR "Ecuador" OR "Estonia" OR "Equatorial Guinea" OR "Fiji" OR "Gabon" OR "Grenada" OR "Iran" OR "Iraq" OR "Jamaica" OR "Jordan" OR "Kazakhstan" OR "Latvia" OR "Lebanon" OR "Libya" OR "Lithuania" OR "Macedonia" OR "Malaysia" OR "Indian Ocean Islands" OR "Maldives" OR “Marshall Islands” OR "Mauritius" OR "Mexico" OR "Montenegro" OR "Namibia" OR "Palau" OR "Panama" OR "Peru" OR "Romania" OR "Russia" OR "Russian Federation" OR "Serbia" OR "Seychelles" OR "Slovakia" OR "Slovak" OR "South Africa" OR "Saint Lucia" OR "Saint Vincent and the Grenadines" OR "Suriname" OR "Thailand" OR "Tonga" OR "Tunisia" OR "Turkey" OR "Turkmenistan" OR "Tuvalu" OR "Venezuela" OR Palestine OR Palestinian OR "low resource" OR "under-resourced" OR "resource poor" OR "under-developed" OR "underdeveloped" OR "developing country" OR "developing countries" OR "developing world" OR "third world" OR "lmic" OR ("low" AND "middle" AND "income"))	
#4 Combined	#1 AND #2 AND #3	183

Global Health (via EBSCOhost)
Search date: 12/16/2024

Concept	Strategy	Results
S1 Pregnancy Terms	DE Pregnancy OR TI Pregnancy OR TI pregnancies OR TI pregnant OR TI gestating OR TI gestational OR TI gestated OR TI gestates OR TI gestate OR TI gestation OR TI maternal OR TI maternity OR AB Pregnancy OR AB pregnancies OR AB pregnant OR AB gestating OR	227,175

	AB gestational OR AB gestated OR AB gestates OR AB gestate OR AB gestation OR AB maternal OR AB maternity	
S2 Rheumatic Heart Disease	TI "Rheumatic Heart Disease" OR TI “rheumatic heart diseases” OR TI “Bouillaud Disease” OR TI “Bouillaud Diseases” OR TI “Bouillaud’s Disease” OR TI “Bouillaud’s Diseases” OR TI “Bouillauds Disease” OR TI “Bouillauds Diseases” OR TI “mitral valve stenosis” OR TI “Mitral valve stenoses” OR TI “mitral stenosis” OR TI “mitral stenoses” OR TI “mitral valve regurgitation” OR TI “mitral regurgitation” OR TI “aortic valve stenosis” OR TI “aortic valve stenoses” OR TI “aortic stenosis” OR TI “aortic stenoses” OR TI “aortic regurgitation” OR TI "Tricuspid Valve Stenosis" OR TI "Tricuspid Valve Stenoses"[tiab] OR TI “tricuspid stenosis” OR TI “tricuspid stenoses” OR TI “tricuspid regurgitation” OR TI "Pulmonary Valve Stenosis" OR TI "Pulmonary Valve Stenoses" OR TI "Pulmonary Stenosis" OR TI "Pulmonary Stenoses" OR TI “pulmonary regurgitation” OR AB "Rheumatic Heart Disease" OR AB “rheumatic heart diseases” OR AB “Bouillaud Disease” OR AB “Bouillaud Diseases” OR AB “Bouillaud’s Disease” OR AB “Bouillaud’s Diseases” OR AB “Bouillauds Disease” OR AB “Bouillauds Diseases” OR AB “mitral valve stenosis” OR AB “Mitral valve stenoses” OR AB “mitral stenosis” OR AB “mitral stenoses” OR AB “mitral valve regurgitation” OR AB “mitral regurgitation” OR AB “aortic valve stenosis” OR AB “aortic valve stenoses” OR AB “aortic stenosis” OR AB “aortic stenoses” OR AB “aortic regurgitation” OR AB "Tricuspid Valve Stenosis" OR AB "Tricuspid Valve Stenoses"[tiab] OR AB “tricuspid stenosis” OR AB “tricuspid stenoses” OR AB “tricuspid regurgitation” OR AB "Pulmonary Valve Stenosis" OR AB "Pulmonary Valve Stenoses" OR AB "Pulmonary Stenosis" OR AB "Pulmonary Stenoses" OR AB “pulmonary regurgitation”	2,632
S3 LMIC Terms	((TI afghanistan OR AB afghanistan) OR (TI afghan* OR AB afghan*) OR (TI afghanistani* OR AB afghanistani*) OR (TI afghani* OR AB afghani*) OR (TI afghanese OR AB afghanese) OR (TI bangladesh OR AB bangladesh) OR (TI bangladeshi* OR AB bangladeshi*) OR (TI benin OR AB benin) OR (TI "edo people" OR AB "edo people") OR (TI "edo person" OR AB "edo person") OR (TI bini OR AB bini) OR (TI "burkina faso" OR AB "burkina faso") OR (TI burkinabe* OR AB burkinabe*) OR (TI burundi OR AB burundi) OR (TI burundian* OR AB burundian*) OR (TI cambodia OR AB cambodia) OR (TI cambodian* OR AB cambodian*) OR (TI "cabo verde" OR AB "cabo verde") OR (TI "cape verdean" OR AB "cape verdean") OR (TI "cape verdeans" OR AB "cape verdeans") OR (TI "cabo verdean" OR AB "cabo verdean") OR (TI "cabo verdeans" OR AB "cabo verdeans") OR (TI "central african republic" OR AB "central african republic") OR (TI "central african" OR AB "central african") OR (TI "central africans" OR AB "central africans") OR (TI chad OR AB chad) OR (TI chadian* OR AB chadian*) OR (TI comoros OR AB comoros) OR (TI comorian* OR AB comorian*) OR (TI "democratic republic of the congo" OR AB "democratic republic of the congo") OR (TI congolese* OR AB congolese*) OR (TI eritrea OR AB eritrea) OR (TI eritrean* OR AB eritrean*) OR (TI ethiopia OR	1,228,764

	AB ethiopia) OR (TI ethiopian* OR AB ethiopian*) OR (TI gambia OR AB gambia) OR (TI gambian* OR AB gambian*) OR (TI guinea OR AB guinea) OR (TI guinean* OR AB guinean*) OR (TI guinea-bissau OR AB guinea-bissau) OR (TI "bissau guinean" OR AB "bissau guinean") OR (TI "bissau guineans" OR AB "bissau guineans") OR (TI bissau-guinean* OR AB bissau-guinean*) OR (TI haiti OR AB haiti) OR (TI haitian* OR AB haitian*) OR (TI kenya OR AB kenya) OR (TI kenyan* OR AB kenyan*) OR (TI "democratic people's republic of korea" OR AB "democratic people's republic of korea") OR (TI "north korean" OR AB "north korean") OR (TI "north korenas" OR AB "north korenas") OR (TI liberia OR AB liberia) OR (TI liberian* OR AB liberian*) OR (TI madagascar OR AB madagascar) OR (TI malagasy* OR AB malagasy*) OR (TI madagascan* OR AB madagascan*) OR (TI malawi OR AB malawi) OR (TI malawian* OR AB malawian*) OR (TI mali OR AB mali) OR (TI malian* OR AB malian*) OR (TI mozambique OR AB mozambique) OR (TI mozambican* OR AB mozambican*) OR (TI myanmar OR AB myanmar) OR (TI "burmese*or myanma*" OR AB "burmese*or myanma*") OR (TI nepal OR AB nepal) OR (TI nepali* OR AB nepali*) OR (TI nepalese OR AB nepalese) OR (TI niger OR AB niger) OR (TI nigerien* OR AB nigerien*) OR (TI rwanda OR AB rwanda) OR (TI rwandan* OR AB rwandan*) OR (TI rwandese OR AB rwandese) OR (TI "sierra leone" OR AB "sierra leone") OR (TI "sierra leonean" OR AB "sierra leonean") OR (TI "sierra leoneans" OR AB "sierra leoneans") OR (TI somalia OR AB somalia) OR (TI somali* OR AB somali*) OR (TI tajikistan OR AB tajikistan) OR (TI tajikstani* OR AB tajikstani*) OR (TI tajik* OR AB tajik*) OR (TI tanzania OR AB tanzania) OR (TI tanzanian* OR AB tanzanian*) OR (TI togo OR AB togo) OR (TI togolese* OR AB togolese*) OR (TI uganda OR AB uganda) OR (TI ugandan* OR AB ugandan*) OR (TI zimbabwe OR AB zimbabwe) OR (TI zimbabwean* OR AB zimbabwean*) OR (TI zimbo* OR AB zimbo*) OR (TI armenia OR AB armenia) OR (TI armenian* OR AB armenian*) OR (TI bhutan OR AB bhutan) OR (TI bhutanese OR AB bhutanese) OR (TI bolivia OR AB bolivia) OR (TI bolivian* OR AB bolivian*) OR (TI cameroon OR AB cameroon) OR (TI cameroonian* OR AB cameroonian*) OR (TI "cape verde" OR AB "cape verde") OR (TI congo OR AB congo) OR (TI "cote d'ivoire" OR AB "cote d'ivoire") OR (TI ivorian* OR AB ivorian*) OR (TI djibouti OR AB djibouti) OR (TI djiboutian* OR AB djiboutian*) OR (TI egypt OR AB egypt) OR (TI egyptian* OR AB egyptian*) OR (TI "el salvador" OR AB "el salvador") OR (TI salvadoran* OR AB salvadoran*) OR (TI "georgia (republic)" OR AB "georgia (republic)") OR (TI georgian* OR AB georgian*) OR (TI ghana OR AB ghana) OR (TI ghanaian* OR AB ghanaian*) OR (TI guatemala OR AB guatemala) OR (TI guatemalan* OR AB guatemalan*) OR (TI guatemalteco* OR AB guatemalteco*) OR (TI guatemalense* OR AB guatemalense*) OR (TI guyana OR AB guyana) OR (TI guyanese OR AB guyanese) OR (TI honduras OR AB honduras) OR (TI honduran* OR AB honduran*) OR (TI indonesia OR AB indonesia) OR (TI indonesian* OR AB indonesian*) OR (TI india OR AB india) OR (TI indian* OR AB indian*) OR (TI kiribati	

	OR AB kiribati) OR (TI gilbertese* OR AB gilbertese*) OR (TI kosovo OR AB kosovo) OR (TI kosovar* OR AB kosovar*) OR (TI kosovan* OR AB kosovan*) OR (TI kyrgyzstan OR AB kyrgyzstan) OR (TI kyrgyzstani* OR AB kyrgyzstani*) OR (TI kirgiz OR AB kirgiz) OR (TI kirghiz OR AB kirghiz) OR (TI kyrgyz OR AB kyrgyz) OR (TI laos OR AB laos) OR (TI laotian* OR AB laotian*) OR (TI lao OR AB lao) OR (TI lesotho OR AB lesotho) OR (TI mosotho* OR AB mosotho*) OR (TI basotho* OR AB basotho*) OR (TI mauritania OR AB mauritania) OR (TI mauritanian* OR AB mauritanian*) OR (TI micronesia OR AB micronesia) OR (TI micronesian* OR AB micronesian*) OR (TI moldova OR AB moldova) OR (TI moldovan* OR AB moldovan*) OR (TI mongolia OR AB mongolia) OR (TI mongolian* OR AB mongolian*) OR (TI morocco OR AB morocco) OR (TI moroccan* OR AB moroccan*) OR (TI nicaragua OR AB nicaragua) OR (TI nicaraguan* OR AB nicaraguan*) OR (TI nigeria OR AB nigeria) OR (TI nigerian* OR AB nigerian*) OR (TI pakistan OR AB pakistan) OR (TI pakistani* OR AB pakistani*) OR (TI "papua new guinea" OR AB "papua new guinea") OR (TI "papua new guinean" OR AB "papua new guinean") OR (TI "papua new guineans" OR AB "papua new guineans") OR (TI paraguay OR AB paraguay) OR (TI paraguayan* OR AB paraguayan*) OR (TI philippines OR AB philippines) OR (TI filipin* OR AB filipin*) OR (TI pinoy* OR AB pinoy*) OR (TI pinay* OR AB pinay*) OR (TI "independent state of samoa" OR AB "independent state of samoa") OR (TI samoan* OR AB samoan*) OR (TI "atlantic islands" OR AB "atlantic islands") OR (TI "sao tome" OR AB "sao tome") OR (TI "sao tomean" OR AB "sao tomean") OR (TI "sao tomeans" OR AB "sao tomeans") OR (TI santomean* OR AB santomean*) OR (TI principe OR AB principe) OR (TI senegal OR AB senegal) OR (TI senegalese* OR AB senegalese*) OR (TI melanesia OR AB melanesia) OR (TI melanesian* OR AB melanesian*) OR (TI "solomon islands" OR AB "solomon islands") OR (TI "solomon islander" OR AB "solomon islander") OR (TI "solomon islanders" OR AB "solomon islanders") OR (TI "sri lanka" OR AB "sri lanka") OR (TI "sri lankan" OR AB "sri lankan") OR (TI "sri lankans" OR AB "sri lankans") OR (TI sinhalese OR AB sinhalese) OR (TI sudan OR AB sudan) OR (TI sudanese OR AB sudanese) OR (TI swaziland OR AB swaziland) OR (TI swazi* OR AB swazi*) OR (TI liswati* OR AB liswati*) OR (TI eswatini OR AB eswatini) OR (TI syria OR AB syria) OR (TI syrian* OR AB syrian*) OR (TI "east timor" OR AB "east timor") OR (TI "east timorese" OR AB "east timorese") OR (TI "timor leste" OR AB "timor leste") OR (TI timorese OR AB timorese) OR (TI ukraine OR AB ukraine) OR (TI ukrainian* OR AB ukrainian*) OR (TI uzbekistan OR AB uzbekistan) OR (TI uzbekistani* OR AB uzbekistani*) OR (TI vanuatu OR AB vanuatu) OR (TI vanuatuan* OR AB vanuatuan*) OR (TI vietnam OR AB vietnam) OR (TI vietnamese* OR AB vietnamese*) OR (TI "middle east" OR AB "middle east") OR (TI "middle eastern" OR AB "middle eastern") OR (TI "west bank" OR AB "west bank") OR (TI gaza OR AB gaza) OR (TI palestinian* OR AB palestinian*) OR (TI gazan* OR AB gazan*) OR (TI yemen OR AB yemen) OR (TI yemeni* OR AB yemeni*) OR (TI zambia OR AB	

	zambia) OR (TI zambian* OR AB zambian*) OR (TI angola OR AB angola) OR (TI angolan* OR AB angolan*) OR (TI albania OR AB albania) OR (TI albanian* OR AB albanian*) OR (TI algeria OR AB algeria) OR (TI algerian* OR AB algerian*) OR (TI argentina OR AB argentina) OR (TI argentine* OR AB argentine*) OR (TI argentinean* OR AB argentinean*) OR (TI argentinian* OR AB argentinian*) OR (TI samoa OR AB samoa) OR (TI samoan* OR AB samoan*) OR (TI azerbaijan OR AB azerbaijan) OR (TI azerbaijani* OR AB azerbaijani*) OR (TI azeri* OR AB azeri*) OR (TI "republic of belarus" OR AB "republic of belarus") OR (TI belarus OR AB belarus) OR (TI belarusian* OR AB belarusian*) OR (TI belize OR AB belize) OR (TI belizean* OR AB belizean*) OR (TI bosnia-herzegovina OR AB bosnia-herzegovina) OR (TI bosnian* OR AB bosnian*) OR (TI botswana OR AB botswana) OR (TI batswana* OR AB batswana*) OR (TI motswana* OR AB motswana*) OR (TI brazil OR AB brazil) OR (TI brazilian* OR AB brazilian*) OR (TI bulgaria OR AB bulgaria) OR (TI bulgarian* OR AB bulgarian*) OR (TI china OR AB china) OR (TI chinese OR AB chinese) OR (TI colombia OR AB colombia) OR (TI colombian* OR AB colombian*) OR (TI "costa rica" OR AB "costa rica") OR (TI "costa rican" OR AB "costa rican") OR (TI "costa ricans" OR AB "costa ricans") OR (TI cuba OR AB cuba) OR (TI cuban* OR AB cuban*) OR (TI dominica OR AB dominica) OR (TI dominican* OR AB dominican*) OR (TI "dominican republic" OR AB "dominican republic") OR (TI ecuador OR AB ecuador) OR (TI ecuadorian* OR AB ecuadorian*) OR (TI "equatorial guinea" OR AB "equatorial guinea") OR (TI equatoguinean* OR AB equatoguinean*) OR (TI "equatorial guinean" OR AB "equatorial guinean") OR (TI "equatorial guineans" OR AB "equatorial guineans") OR (TI fiji OR AB fiji) OR (TI fijian* OR AB fijian*) OR (TI gabon OR AB gabon) OR (TI gabonese OR AB gabonese) OR (TI gabonaise OR AB gabonaise) OR (TI grenada OR AB grenada) OR (TI grenadian* OR AB grenadian*) OR (TI iran OR AB iran) OR (TI iranian* OR AB iranian*) OR (TI iraq OR AB iraq) OR (TI iraqi* OR AB iraqi*) OR (TI jamaica OR AB jamaica) OR (TI jamaican* OR AB jamaican*) OR (TI jordan OR AB jordan) OR (TI jordanian* OR AB jordanian*) OR (TI kazakhstan OR AB kazakhstan) OR (TI kazakhstani* OR AB kazakhstani*) OR (TI lebanon OR AB lebanon) OR (TI lebanese OR AB lebanese) OR (TI libya OR AB libya) OR (TI libyan* OR AB libyan*) OR (TI macedonia OR AB macedonia) OR (TI macedonian* OR AB macedonian*) OR (TI malaysia OR AB malaysia) OR (TI malaysian* OR AB malaysian*) OR (TI "indian ocean islands" OR AB "indian ocean islands") OR (TI maldives OR AB maldives) OR (TI maldivian* OR AB maldivian*) OR (TI "marshall islands" OR AB "marshall islands") OR (TI marshallese OR AB marshallese) OR (TI mauritius OR AB mauritius) OR (TI mauritian* OR AB mauritian*) OR (TI mexico OR AB mexico) OR (TI mexican* OR AB mexican*) OR (TI montenegro OR AB montenegro) OR (TI montenegrin* OR AB montenegrin*) OR (TI namibia OR AB namibia) OR (TI namibian* OR AB namibian*) OR (TI palau OR AB palau) OR (TI palauan* OR AB palauan*) OR (TI panama OR AB panama) OR (TI panamanian* OR AB panamanian*)	

	OR (TI peru OR AB peru) OR (TI peruvian* OR AB peruvian*) OR (TI romania OR AB romania) OR (TI romanian* OR AB romanian*) OR (TI russia OR AB russia) OR (TI russian* OR AB russian*) OR (TI serbia OR AB serbia) OR (TI serbian* OR AB serbian*) OR (TI seychelles OR AB seychelles) OR (TI seychellois* OR AB seychellois*) OR (TI seselwa* OR AB seselwa*) OR (TI "south africa" OR AB "south africa") OR (TI "south african" OR AB "south african") OR (TI "south africans" OR AB "south africans") OR (TI "saint lucia" OR AB "saint lucia") OR (TI "saint lucian" OR AB "saint lucian") OR (TI "saint lucians" OR AB "saint lucians") OR (TI "saint vincent and the grenadinesor "st vincent"" OR AB "saint vincent and the grenadinesor "st vincent"") OR (TI "saint vincent" OR AB "saint vincent") OR (TI grenadines OR AB grenadines) OR (TI "west indies" OR AB "west indies") OR (TI vincentian* OR AB vincentian*) OR (TI grenadinian* OR AB grenadinian*) OR (TI vincy OR AB vincy) OR (TI suriname OR AB suriname) OR (TI surinamese* OR AB surinamese*) OR (TI thailand OR AB thailand) OR (TI thai OR AB thai) OR (TI tonga OR AB tonga) OR (TI tongan* OR AB tongan*) OR (TI tunisia OR AB tunisia) OR (TI tunisian* OR AB tunisian*) OR (TI turkey OR AB turkey) OR (TI turkish OR AB turkish) OR (TI turk OR AB turk) OR (TI turkmenistan OR AB turkmenistan) OR (TI turkmenistani* OR AB turkmenistani*) OR (TI turkmen* OR AB turkmen*) OR (TI turkmenian* OR AB turkmenian*) OR (TI tuvalu OR AB tuvalu) OR (TI tuvaluan* OR AB tuvaluan*) OR (TI venezuela OR AB venezuela) OR (TI venezuelan* OR AB venezuelan*) OR (TI "low resource" OR AB "low resource") OR (TI under-resourced OR AB under-resourced) OR (TI underresourced OR AB underresourced) OR (TI "resource poor" OR AB "resource poor") OR (TI "resource limited" OR AB "resource limited") OR (TI under-developed OR AB under-developed) OR (TI underdeveloped OR AB underdeveloped) OR (TI "developing country" OR AB "developing country") OR (TI "developing countries" OR AB "developing countries") OR (TI "developing world" OR AB "developing world") OR (TI "third world" OR AB "third world") OR (TI lmic OR AB lmic) OR (TI lmics OR AB lmics) OR (TI "central America" OR AB "central America") OR (TI "central american" OR AB "central american") OR (TI "south America" OR AB "south America") OR (TI "south American" OR AB "south American") OR (TI "southeast Asia" OR AB "southeast Asia") OR (TI "southeast Asian" OR AB "southeast Asian") OR (TI "pacific islands" OR AB "pacific islands") OR (TI "pacific islander" OR AB "pacific islander") OR (TI "pacific islanders" OR AB "pacific islanders") OR (TI sub-saharan OR AB sub-saharan) OR (TI Caribbean OR AB Caribbean) OR (TI "latin America" OR AB "latin America") OR (TI "latin American" OR AB "latin American") OR (TI oceania OR AB oceania)) OR ((TI low OR AB low) N2 (TI income OR AB income)) OR ((TI middle OR AB middle) N2 (TI income OR AB income))	
S4 Combined	S1 AND S2 AND S3	59

Global Index Medicus (via World Health Organization)
Search date: 12/16/2024

Concept	Strategy	Results
#1 Pregnancy Terms AND Rheumatic Heart Disease	(Pregnancy OR pregnancies OR pregnant OR gestating OR gestational OR gestated OR gestates OR gestate OR gestation OR maternal OR maternity) AND (“Rheumatic Heart Disease" OR “rheumatic heart diseases” OR “Bouillaud Disease” OR “Bouillaud Diseases” OR “Bouillaud’s Disease” OR “Bouillaud’s Diseases” OR “Bouillauds Disease” OR “Bouillauds Diseases” OR “mitral valve stenosis” OR “Mitral valve stenoses” OR “mitral stenosis” OR “mitral stenoses” OR “mitral valve regurgitation” OR “mitral regurgitation” OR “aortic valve stenosis” OR “aortic valve stenoses” OR “aortic stenosis” OR “aortic stenoses” OR “aortic regurgitation” OR "Tricuspid Valve Stenosis" OR "Tricuspid Valve Stenoses" OR “tricuspid stenosis” OR “tricuspid stenoses” OR “tricuspid regurgitation” OR "Pulmonary Valve Stenosis" OR "Pulmonary Valve Stenoses" OR "Pulmonary Stenosis" OR "Pulmonary Stenoses" OR “pulmonary regurgitation”)	426

Supplemental Table 2. Reasons for exclusion

**Reason**	**Number of studies**
Study location (not located in Sub-Saharan Africa)	23
Review	18
Does not address outcomes of interest	16
Case report	8
Not correct population (does not include pregnant individuals)	7
Combined study with < 60% of individuals with RHD	6
Guideline, recommendation or practice management	4
Not rheumatic heart disease	3
Unable to locate article	2
Editorial, letter or comment	2
Combined country data	1
Study ended before 2000	1
**Total**	**91**

Supplemental Table 3. Risk of bias for case series using the JBI critical appraisal checklist

Author	Q1	Q2	Q3	Q4	Q5	Q6	Q7	Q8	Q9	Q10	Overall appraisal	Notes
**Desai et al.**[1]	Y	U	Y	Y	Y	Y	Y	Y	Y	N	Include	Outcomes are not clearly described or defined in the methods section. Unclear reporting of multivariate analysis.
**Diao et al.**[2]	Y	U	U	Y	Y	Y	Y	Y	N	Y	Include	
**Gebremedhin et al.**[3]	Y	Y	U	Y	Y	Y	Y	U	Y	Y	Include	"Unfavorable" outcomes are not clearly described or defined in the methods section. Not peer reviewed.
**Hailu et al.**[4]	Y	Y	Y	Y	Y	Y	Y	Y	Y	Y	Include	
**Nqayana et al.**[5]	Y	Y	Y	Y	Y	Y	Y	Y	U	Y	Include	
**Poli et al.**[6]	Y	Y	Y	Y	U	Y	Y	Y	N	Y	Include	
**Soma-Pillay et al.**[7]	Y	U	U	Y	Y	Y	Y	Y	Y	N/A	Include	Outcomes are not clearly described or defined in the methods section.

Options for Q1-10 include: Yes (Y), No (N), Unclear (U) and N/A
Options for overall appraisal include: Include, Exclude and Seek further info
Q1: Were there clear criteria for inclusion in the case series? Q2: Was the condition measured in a standard, reliable way for all participants included in the case series? Q3: Were valid methods used for identification of the condition for all participants included in the case series? Q4: Did the case series have consecutive inclusion of participants? Q5: Did the case series have complete inclusion of participants? Q6: Was there clear reporting of the demographics of the participants in the study? Q7: Was there clear reporting of clinical information of the participants? Q8: Were the outcomes or follow up results of cases clearly reported? Q9: Was there clear reporting of the presenting site(s)/clinic(s) demographic information? Q10: Was statistical analysis appropriate?
Questions 1, 4, and 5 are related to "bias in selection of participants into the study"; Questions 2 and 3 address "bias in measurement of outcomes"; questions 6 and 7 address "bias in selection of the reported results"; and question 8 addresses "bias due to missing data."[8]

Supplemental Table 4. Risk of bias for cohort studies using the JBI critical appraisal checklist

Author	Q1	Q2	Q3	Q4	Q5	Q6	Q7	Q8	Q9	Q10	Q11	Overall appraisal	Notes
**Beaton et al.**[9]	Y	Y	Y	Y	U	Y	Y	Y	Y	N/A	Y	Include	

Options for Q1-11 include: Yes (Y), No (N), Unclear (U) and N/A
Options for overall appraisal include: Include, Exclude and Seek further info
Q1: Were the two groups similar and recruited from the same population? Q2: Were the exposures measured similarly to assign people to both exposed and unexposed groups? Q3: Was the exposure measured in a valid and reliable way? Q4: Were confounding factors identified? Q5: Were strategies to deal with confounding factors stated? Q6: Were the groups/participants free of the outcome at the start of the study (or at the moment of exposure)? Q7: Were the outcomes measured in a valid and reliable way? Q8: Was the follow up time reported and sufficient to be long enough for outcomes to occur? Q9: Was follow up complete, and if not, were the reasons to loss to follow up described and explored? Q10: Were strategies to address incomplete follow up utilized? Q11: Was appropriate statistical analysis used?

Supplemental Table 5. Risk of bias for case-control studies using the JBI critical appraisal checklist

Author	Q1	Q2	Q3	Q4	Q5	Q6	Q7	Q8	Q9	Q10	Overall appraisal	Notes
**Lumsden et al.** [10]	Y	Y	Y	Y	Y	Y	Y	Y	Y	Y	Include	
Options for Q1-10 include: Yes (Y), No (N), Unclear (U) and N/A<br>Options for overall appraisal include: Include, Exclude and Seek further info<br>Q1: Were the groups comparable other than the presence of disease in cases or the absence of disease in controls? Q2: Were cases and controls matched appropriately? Q3: Were the same criteria used for identification of cases and controls? Q4: Was exposure measured in a standard, valid and reliable way? Q5: Was exposure measured in the same way for cases and controls? Q6: Were confounding factors identified? Q7: Were strategies to deal with confounding factors stated? Q8: Were outcomes assessed in a standard, valid and reliable way for cases and controls? Q9: Was the exposure period of interest long enough to be meaningful? Q10: Was appropriate statistical analysis used?												

Supplemental References

1. Desai DK, Adanlawo M. Mitral stenosis in pregnancy: a four-year experience at King Edward VIII Hospital, Durban, South Africa. *Br J Obstet Gynaecol*. 2000;107:953-958.

2. Diao M. Pregnancy in women with heart disease in sub-Saharan Africa. *Archives of Cardiovascular Disease*. 2011;104:370-374. doi:10.1016/j.acvd.2011.04.001

3. Gebremedhin Y, Guteta S, Melese B, Bekele E. Predictors of maternal and fetal outcomes of pregnant women with mitral stenosis. Published online 2022. doi:10.1101/2022.08.04.22278407

4. Hailu A, Yeman A, Abate E, Teka H, Berhane H, Whe A. Management and outcome of severe pulmonary hypertension in pregnancy: Experience from a university hospital in northern Ethiopia. *Ethiop Med J*. 2019;57.

5. Nqayana T, Moodley J, Naidoo D. Cardiac disease in pregnancy. *Cardiovascular Journal of Africa*. 2008;19(3).

6. Poli PA, Orang'o EO, Mwangi A, Barasa FA. Factors Related to Maternal Adverse Outcomes in Pregnant Women with Cardiac Disease in Low-resource Settings. *European Cardiology Review*. 2020;15(e68). doi:10.15420

7. Soma-Pillay P, MacDonald AP, Mathivha TM, Bakker JL, Mackintosh MO. Cardiac disease in pregnancy: a 4-year audit at Pretoria Academic Hospital. *S Afr Med J*. 2008;98(7):553-556.

8. Munn Z, Barker T, Moola S, Tufanaru C, Stern C, McArthur A, Stephenson M, Aromataris E. Methodological quality of case series studies, JBI Evidence Synthesis, doi: 10.11124/JBISRIR-D-19-00099.

9. Beaton A, Okello E, Scheel A, et al. Impact of heart disease on maternal, fetal and neonatal outcomes in a low-resource setting. *Heart (British Cardiac Society)*. 2018;105(10):755. doi:10.1136/heartjnl-2018-313810

10. Lumsden R, Barasa F, Park LP, et al. High Burden of Cardiac Disease in Pregnancy at a National Referral Hospital in Western Kenya. *gh*. 2020;15(1):10. doi:10.5334/gh.404
